# Automated fluorine-18 radiolabeling via an alkyne–azide cycloaddition reaction on a dual peptide-functionalized liposome surface for *in vivo* PET imaging

**DOI:** 10.3389/fphar.2025.1566257

**Published:** 2025-04-28

**Authors:** Marco Iannone, Marcelo Kravicz, Paolo Rainone, Antonia I. Antoniou, Stefano Stucchi, Silvia Valtorta, Arianna Amenta, Elia Anna Turolla, Sara Pellegrino, Daniele Passarella, Elisa Vino, Sergio Todde, Francesca Re, Pierfausto Seneci, Rosa Maria Moresco

**Affiliations:** ^1^ Tecnomed Foundation, University of Milano-Bicocca, Monza, Italy; ^2^ School of Medicine and Surgery, University of Milano-Bicocca, Monza, Italy; ^3^ nstitute of Bioimaging and Biological Complex Systems (IBSBC), National Research Council (CNR), Segrate, Italy; ^4^ Nuclear Medicine Department, San Raffaele Scientific Institute IRCCS, Milan, Italy; ^5^ Department of Chemistry, University of Milan, Milan, Italy; ^6^ National Biodiversity Future Center (NBFC), Palermo, Italy; ^7^ GBM‐BI‐TRACE (GlioBlastoMa‐BIcocca‐TRAnslational‐CEnter), University of Milano‐Bicocca, Monza, Italy; ^8^ Department of Pharmaceutical Sciences, University of Milan, Milan, Italy

**Keywords:** liposome, radiolabeling, copper(I)-catalyzed cycloaddition click, copper-free cycloaddition, positron emission tomography imaging, glioma, metalloproteases

## Abstract

**Introduction:**

Labeled nanoparticles can be monitored in the body using positron emission tomography (PET) imaging, providing real-time insights into their pharmacokinetics and biodistribution. In the present work, liposomes are labeled with the radionuclide fluorine-18, exploiting a “surface radiolabeling” approach.

**Methods:**

Two alkyne-dioleoylphosphatidylethanolamine (DOPE) constructs are embedded within the bulk of the liposome bilayer, which is composed of cholesterol (Ch) and sphingomyelin (SM), and radiolabeling is performed via either a copper(I)-catalyzed cycloaddition “click” reaction (CuAAC) or a cyclooctyne-driven copper-free “click” reaction (CyOctC) modality, using a suitable fluorine-18 labeled azide, obtaining good results in terms of yield, purity, stability, and automation of the entire radiosynthesis process. In addition, radiolabeling is also performed on liposome formulations functionalized with 1) a peptide derived from the receptor-binding domain of apolipoprotein E (mApoE) and 2) a metalloproteinase (MMP)-sensitive lipopeptide (MSLP). The in vivo uptake of these liposomes is evaluated in an orthotopic glioma mouse model (Gli36ΔEGFR cell line) using PET/computed tomography (CT).

**Results and discussion:**

The results demonstrate a higher tumor/background ratio, a faster clearance rate, and a lower uptake in healthy brain tissue and peripheral regions for mApoE- and MSLP-functionalized liposomes than for non-functionalized liposomes, prompting further characterization. On the contrary, radiolabeled liposome uptake is higher in the majority of peripheral organs for non-functionalized liposomes. Hence, fluorine-18-labeled liposomes can be reliably used for in vivo PET tracking of multifunctionalized nanoparticles, enabling effective investigation of their potential as drug delivery systems.

## Highlights

• This study presents the first automated fluorine-18 radiolabeling on a liposome surface by cycloaddition.
• Radiolabeling was performed on functionalized liposomes containing mApoE and a metalloproteinase (MMP)-sensitive lipopeptide (MSLP).
• The resulting radiolabeled liposome formulation is suitable for future preclinical studies.

## 1 Introduction

A large number of drugs are characterized by limited brain penetrability. The development of advanced delivery systems, such as nanoparticles, may help overcome the blood–brain barrier (BBB) and increase brain accessibility (Spinelli et al., 2019). These systems, which are often engineered to enhance brain accessibility, can mask the physicochemical properties of encapsulated or associated drugs to improve their delivery to the brain parenchyma (Dong, 2018). Nanoparticle functionalization can also be used to develop controlled agents capable of releasing drugs based on the pathological biochemical environment in the target region (Antoniou et al., 2021; Wang and Kohane, 2017). Among different nanoparticles, liposomes show several advantages, including synthetic flexibility, biodegradability, biocompatibility, and low immunogenicity and toxicity. For these reasons, a wide variety of liposome-based formulations have been approved by regulatory agencies as drug delivery systems (Liu et al., 2022). We previously developed an innovative dual-functionalized liposome designed to increase drug availability in brain regions characterized by an inflammatory milieu. In particular, we modified the liposome surface with a peptide derived from the binding domain of apolipoprotein E (mApoE), which is known to increase brain penetration (Bana et al., 2014), and with a metalloproteinase (MMP)-sensitive lipopeptide (MSLP) to confer an MMP-dependent drug release. These liposomes successfully crossed the BBB *in vitro* and efficiently released an encapsulated fluorescein dye when exposed to MMP-2 and MMP-9, demonstrating their potential for delivering payloads to the brain (Giofrè et al., 2022). Therefore, an automated procedure for the radiolabeling of such functionalized liposomes was developed, using positron emission tomography (PET) imaging to assess whether the BBB penetrability profile of liposomes is maintained *in vivo*.

Single-photon emission computed tomography (SPECT) and PET enable non-invasive tracking of radiolabeled nanoparticles in the body, providing an *in vivo* understanding of their biodistribution properties at the organ level (Man et al., 2019). Glioma was selected due to the following reasons: 1) MMPs are overexpressed not only in microglia but also in cancer cells, thus favoring the controlled delivery effect of the MMP-sensitive lipopeptide (Hagemann et al., 2012; Markovic et al., 2005); 2) disease modifying therapy for glioma still represents an unmet medical need, and poor brain penetration of drugs represents a significant limitation in drug development; and 3) the comparison between tumor and normal brain parenchyma distribution may provide information on the potential lesion selectivity of the drug effect. As a model for the evaluation of the *in vivo* brain distribution of liposomes, already characterized temozolomide-resistant orthotopic EGFR-mutated mouse glioma models were selected (Valtorta et al., 2021). Moreover, the selected cell line (data not shown) expresses different MMPs, including MMP-9. For *in vivo* kinetic studies, the choice of the radionuclide is crucial, as the radionuclide half-life and the (radio)chemistry involved in the labeling process may affect nanoparticle shape and the eventual translatability to the clinic. Radiolabeling of nanoparticles may be performed using different radionuclides and radiolabeling approaches that include passive encapsulation, membrane labeling, and remote labeling with lipophilic chelators, ionophores, or surface chelators (Low et al., 2023). Among the various available strategies, we selected the “surface radiolabeling” technique, in which a radionuclide is linked to the liposome’s surface (Man et al., 2019), rather than other approaches that involve radiometal internalization within the lipid bilayer. This method is easy to implement and represents an attractive strategy that has been broadly applied using different radiometals (Pérez-Medina et al., 2014; Mougin-Degraef et al., 2007; Varga et al., 2017; Luo et al., 2017; Malinge et al., 2017; Jensen et al., 2018; Helbok et al., 2010). Focusing on PET radionuclides, despite the advantage of long-term monitoring of radioactivity distribution within organs, a general problem in the use of medium–long half-life radiometals, such as zirconium-89 (T_1/2_ = 78.4 h) or copper-64 (T_1/2_ = 12.7 h), is represented by the small number of facilities where the necessary solid/liquid target technology required for their production is available. Due to its favorable physical characteristics (T_1/2_ = 109.8 min, short positron range) and availability, fluorine-18 is the “workhorse” in PET imaging to date (Sanchez-Crespo, 2013). Despite its relatively short half-life, which does not allow long kinetic studies, radiolabeling with fluorine-18 may be sufficient for a proof-of-concept study focused on tumor access of nanoparticles and the tumor-to-normal brain distribution (Okua et al., 2011). In this study, we developed a fully automated method for the “surface” radiolabeling with fluorine-18 via either copper(I)-catalyzed cycloaddition (CuAAC) or via cyclooctyne-driven copper-free (CyOctC) “click” chemistry, using a suitable fluorine-18-labeled azide (Kim and Koo, 2019). The entire radiosynthetic procedure, from [^18^F]fluoroazide formation to radiolabeling and purification of liposomes, was fully automated, yielding the desired products with radiochemical yields and purity levels potentially useful for clinical studies in humans. Finally, the radiolabeled functionalized (with mApoE and an MMP-sensitive peptide) and non-functionalized liposomes were evaluated to compare their penetration and tumor selectivity properties in the abovementioned orthotopic mouse glioma model.

## 2 Materials and methods

### 2.1 General methods—experimental synthetic procedures

All reagents and dry solvents used in the present work were commercially available and were purchased from Sigma-Aldrich (Taufkirchen, Germany) or TCI Europe N.V. (Zwijndrecht, Belgium) and used without further purification. Reactions were carried out under a dry nitrogen atmosphere in pre-flamed glassware when required. Anhydrous Na_2_SO_4_ was used to dry the solutions, and the solvents were then routinely removed at approximately 40°C under reduced pressure using a rotary vacuum evaporator. Flash column chromatography (FCC) was performed on Merck silica gel 60 (240–400 mesh, Darmstadt, Germany), and analytical thin-layer chromatography (TLC) was performed on Merck silica gel 60F254 (0.2 mm film, Darmstadt, Germany) pre-coated on aluminum foil. Spots on the TLC plates were visualized using UV light at 254 nm and by staining the TLC plates with a solution of cerium molybdate (Hanessian’s Stain). ^1^H NMR spectra were obtained at 400.15 MHz, and ^13^C NMR spectra were obtained at 100.63 MHz, using a Bruker DRX-400 spectrometer (Billerica, United States) in the indicated solvents. Chemical shifts (*δ*) are shown in parts per million (ppm) downfield from tetramethylsilane (TMS), and coupling constants (*J*) are reported in Hertz. Electron spray ionization (ESI)–high-resolution mass spectrometry (HRMS) spectra were recorded using a Waters Q-ToF SYNAPT G2-Si HDMS 8K mass spectrometer (Milford, United States).

#### 2.1.1 Synthesis of 4-nitrophenyl prop-2-yn-1-yl carbonate (**1**)

To an ice-cold stirred solution of propargyl alcohol (0.3 mL, 5.0 mmol) and Et_3_N (0.8 mL, 5.75 mmol) in tetrahydrofuran (THF) (10 mL), a solution of *p*-nitrophenyl chloroformate (1.11 g, 5.5 mmol) in THF (5.0 mL) was added dropwise over 30 min. The reaction mixture was stirred for another 12 h at room temperature (rt) and quenched with 20% aqueous NH_4_Cl (5.0 mL). THF was evaporated under reduced pressure, and the residue was extracted with EtOAc (3 × 10 mL). The combined organic phases were washed with brine (15 mL), dried over Na_2_SO_4_, and evaporated under reduced pressure. The oily residue thus obtained was triturated with *n*-hexane and refrigerated overnight. The yellow precipitate was filtered under a vacuum and dried to obtain pure target **1** (906 mg) in 82% yield. ^1^H NMR (400 MHz, CDCl_3_, δ): 8.29 (d, *J* = 9.2 Hz, 2H; H-10 and H-12), 7.41 (d, *J* = 9.2 Hz, 2H; H-9 and H-13), 4.88 (d, J = 2.4 Hz, 2H; H-2), and 2.62 (t, *J* = 2.4 Hz, 1H; H-4); ^13^C NMR (100 MHz, CDCl_3_, δ): 155.4 (C8), 152.1 (C11), 145.6 (C5), 125.4 (C10 and C12), 121.8 (C9 and C13), 76.8 (C1), 76.1 (C4), and 56.6 (C2).

#### 2.1.2 Synthesis of [(1R,8S,9S)-bicyclo (6.1.0)non-4-yn-9-yl]methyl 4-nitrophenyl carbonate (**2**)

To a stirred solution of BCN-OH (32 mg, 0.213 mmol) in DCM (5 mL), pyridine (43 μL, 0.532 mmol) and *p*-nitrophenyl chloroformate (53 mg, 0.266 mmol) were sequentially added. After stirring for 15 min at rt, the reaction mixture was quenched with saturated aqueous NH_4_Cl (5 mL) and extracted with DCM (3 × 5 mL). The organic layer was dried over Na_2_SO_4_ and concentrated *in vacuo*. The residue was purified using FCC to obtain, upon solvent removal, pure target carbonate **2** (35.6 mg, 53%) as a colorless oil. ^1^H NMR (400 MHz, CDCl_3_, δ): 8.27 (d, *J* = 9.2 Hz, 2H; H-17 and H-19), 7.38 (d, *J* = 9.2 Hz, 2H; H-16 and H-20), 4.40 (d, *J* = 8.3 Hz, 2H; H-14), 2.36–2.22 (m, 6H; H-7a, H-8, H-11 and H-12a) 1.65–1.54 (m, 2H; H-7b and H-12b), 1.53–1.45 (m, 1H; H-13), and 1.09–1.01 (m, 2H; H-5 and H-6); ^13^C NMR (100 MHz, CDCl_3_, δ): 155.7 (C15), 152.7 (C18), 145.5 (C3), 125.4 (C17 and C19), 121.9 (C16 and C20), 98.8 (C9 and C10), 68.1 (C14), 29.1 (C12–C7), 21.5 (C8 and C11), 20.6 (C5 and C6), and 17.4 (C13).

#### 2.1.3 Synthesis of DOPE carbamate derivatives m_A_-DOPE and m_CA_-DOPE

Et_3_N (24 mL, 0.17 mmol) was added at 0°C to a stirred solution of either **1** (16.8 mg, 0.076 mmol) or **2** (24 mg, 0.076 mmol) and dioleoylphosphatidylethanolamine (DOPE) (50 mg, 0.068 mmol) in DCM/DMF (2:1, 2 mL). After warming and stirring for 12 h at rt, the mixture was concentrated under reduced pressure; diluted with DCM (20 mL); washed sequentially with 10% aqueous citric acid (5 mL), water (5 mL), and brine (5 mL); dried over Na_2_SO_4_; and concentrated under reduced pressure. The oily residue was purified using FCC to provide, upon solvent removal, either pure target m_A_-DOPE (50.5 mg, 90%) or pure target m_CA_-DOPE (49.4, 79%), both as colorless oils.

*m*_*A*_ *-DOPE*: ^1^H NMR (400 MHz, DMSO-*d*_6_, δ): 7.86 (br s, 1H; NH), 5.32 (t, *J* = 4.7 Hz, 4H; =CH oleoyl), 5.09–5.02 (m, 1H; CH glycerol), 4.58 (d, *J* = 2.4 Hz, 1H; CH_2_-a glycerol), 4.28 (dd, *J* = 12.0, 3.1 Hz, 1H; CH_2_-b glycerol), 4.11–4.02 (m, 2H; CH_2_ glycerol), 3.75–3.58 (m, 4H; CH_2_CH_2_NH), 3.44 (t, *J* = 2.4 Hz, 1H; ≡CH), 3.12 (q, *J* = 5.6 Hz, 2H; CH_2_ propargyl), 2.30–2.23 (m, 4H; CH_2_CO oleoyl), 1.98 (q, *J* = 6.7 Hz, 8H; CH_2_CH = CH oleoyl), 1.54–1.46 (m, 4H; CH_2_CH_2_CO oleoyl), 1.30–1.21 (m, 40H; CH_2_ oleoyl), and 0.86 (t, *J* = 6.7 Hz, 6H; CH_3_ oleoyl); ^13^C NMR (100 MHz, DMSO-*d*_6_, δ): 172.5 (CO oleoyl), 172.2 (CO oleoyl), 155.2 (CO propargyl), 129.6 (=CH), 129.5 (=CH), 79.6 (≡C), 70.5 (≡CH), [62.4, 51.3, 33.6, 33.4, 31.2, 29.1, 28.8, 28.7, 28.6, 28.5, 28.4, 26.6, 24.4, 22.1 (CH_2_ oleoyl)], and 13.9 (CH_3_ oleoyl); HRMS (ESI) *m/z*: [M]^-^ calculated for C_45_H_79_NO_10_P^−^, 824.4542; found, 824.5444.

*m*_*CA*_ *-DOPE*: ^1^H NMR (400 MHz, CDCl_3_, δ): 11.97 (s, 1H, N*H*), 5.38–5.27 (m, 3H; =CH oleoyl), 5.21 (br s, 1H; =CH oleoyl), 4.40–4.09 (m, 4H; CH glycerol, CH_2_ glycerol), 4.06–3.93 (m, 2H; CH_2_ glycerol, CH_2_-a), 3.52–3.39 (m, 1H; CH_2_-b), 3.16–3.05 (m, 4H; CH_2_CH_2_NH), 2.36–2.14 (m, 8H; C*H*_*2*_ cyclooctyne), 2.04–1.95 (m, 6H; CH cyclooctyne, CH_2_ oleoyl), 1.64–1.51 (m, 5H; CH_2_CO oleoyl, CH cyclopropyl), 1.38 (t, *J* = 7.2 Hz, 8H; CH_2_CH = CH oleoyl), 1.34–1.19 (m, 40H; CH_2_ oleoyl), and 0.87 (t, *J* = 7.0 Hz, 6H; CH_3_ oleoyl); ^13^C NMR (100 MHz, CDCl_3_, δ): 173.6 (CO oleoyl), 130.2 (CO), 129.8 (=CH), 129.7 (=CH), 98.9(≡C), 70.6 (CH glycerol), [63.0, 34.4, 34.2, 32.0, 29.9, 29.9, 29.7, 29.5, 29.4, 27.4, 25.1, 25.0, 22.8, 21.5, 20.4, 17.9 (CH_2_ glycerol, CH_2_ oleoyl)], and 14.2 (CH_3_ oleoyl); HRMS (ESI) *m/z*: [M]^-^ calculated for C_52_H_89_NO_10_P^−^, 918.6224; found, 918.6235.

### 2.2 Liposome formulation

Lipid stock solutions of cholesterol (Ch), sphingomyelin (SM), DSPE-PEG-2k-Mal, m_A_-DOPE, and m_CA_-DOPE were prepared in chloroform (CH_3_Cl) and methanol (MeOH) according to the following ratios: Ch (20 mg mL^−1^, 51.7 mM) and DSPE-PEG-2k-Mal (12.3 mg mL^−1^, 4.2 mM), CH_3_Cl: MeOH 100:0; SM, CH_3_Cl: MeOH 87:13; m_A_-DOPE (5.6 mg mL^−1^, 6.7 mM), CH_3_Cl: MeOH 100:0; m_CA_-DOPE, CH_3_Cl: MeOH 90:10. An MSLP stock was prepared in MeOH (0.2 mg mL^−1^), while an mApoE stock was prepared in ultrapure water (1 mg mL^−1^). Liposomes containing m_A_-DOPE (m_A_-Lip) 1, 2, 5, and 10 mol% were prepared by combining Ch, SM, and m_A_-DOPE in a round-bottom flask containing stirred CH_3_Cl (18 mL). After removing the organic solvent using a rotary vacuum pump (Rotavapor^®^ R-300, Büchi, Sankt Gallen, Switzerland) at 25°C, 340 mbar, 85 rpm, and 20 min, the resulting lipid film was flushed with nitrogen. Liposomes were formed by lipid film hydration with ultrapure water to a final concentration of 2 and 10 mM total lipid (L_t_), respectively, at 65°C for 30 min, followed by sonication amplitude 30, continuous mode, tip diameter 1/8″, 20 min (Vibra Cell VCX-130, Sonics, Newtown, United States). A liposomal formulation m_CA_-Lip containing m_CA_-DOPE 5 mol% was prepared similarly. Liposomes containing MSLP and DSPE-PEG-2k-Mal (m_A_-Lip-MSLP-Mal and m_CA_-Lip-MSLP-Mal) as precursors for mApoE functionalization were prepared according to the previous procedure (Giofrè et al., 2022; Rodà et al., 2023) with an additional 1,840 µL of MSLP during film formation. The functionalization of m_A_-Lip-MSLP-Mal and m_CA_-Lip-MSLP-Mal with mApoE was carried out by adding the mApoE peptide stock (33 µL) to a 1,000-µL m_CA_-Lip-MSLP-Mal formulation in a mol ratio of 10:1 DSPE-PEG-2k-Mal: mApoE, and the reaction was carried out overnight (Rodà et al., 2023).

### 2.3 Liposome characterization

The mean particle size, polydispersity index (PDI), and ζ-potential were measured by dynamic light scattering (DLS) and interferometric Doppler velocimetry (Brookhaven Instruments Corporation, Holtsville, United States, equipped with a ZetaPALS device) (Re et al., 2024). Nanoparticle concentration was determined using NanoSight (NS 300, Malvern, United Kingdom) at 25°C and analyzed using nanoparticle tracking analysis (NTA). L_t_ was estimated using a colorimetric determination of phospholipids using the Stewart assay (Stewart, 1980; Mourtas et al., 2008).

### 2.4 CuAAC reaction on the surface of m_A_-Lip and m_A_-Lip-MSLP-mApoE

**FIGURE 1:**
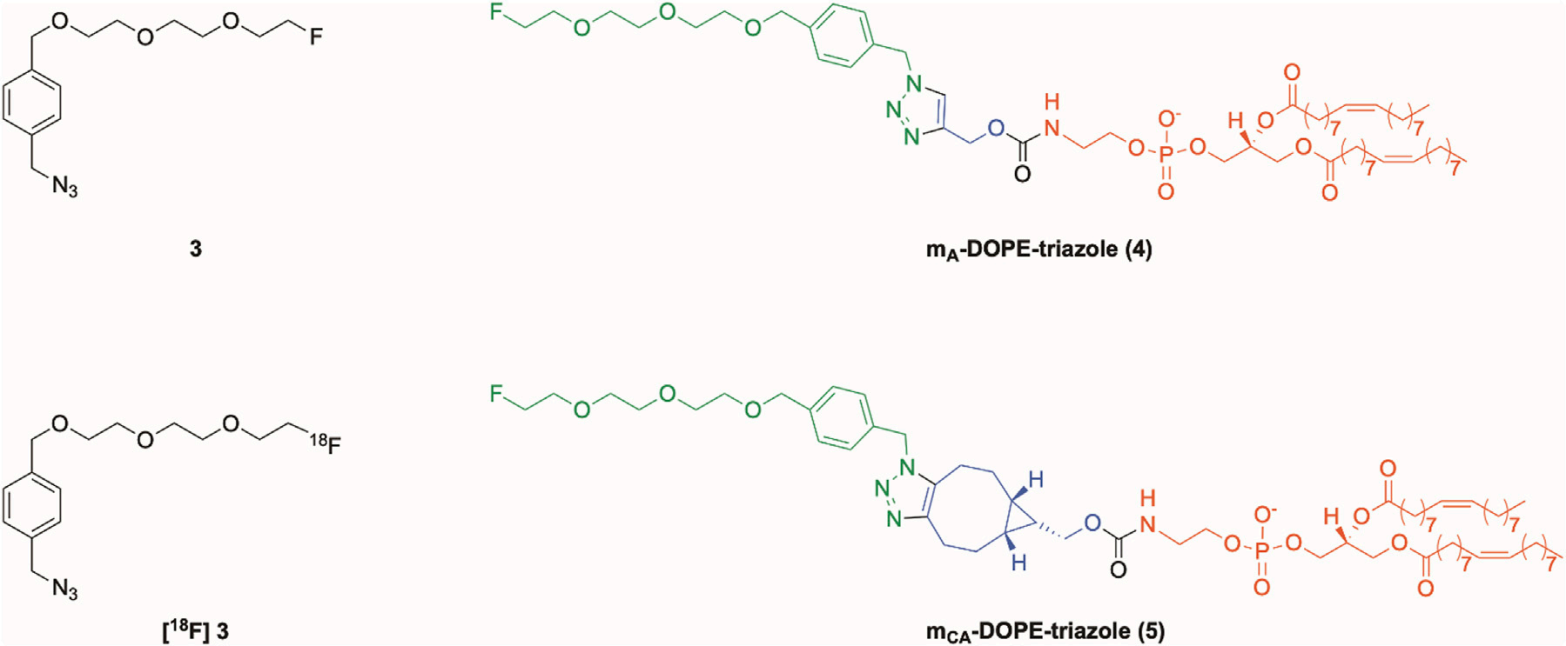
Structures of non-radioactively labeled [^19^F]fluoroazide (**3**), its fluorine-18-labeled counterpart (**[** ^**18**^ **F]3**), triazole obtained by the CuAAC reaction between **3** and m_A_-DOPE (**4**), and triazole obtained by “the copper-free” cycloaddition reaction between **3** and m_CA_-DOPE (**5**).

**SCHEME 1:**
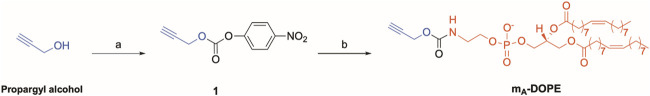
Synthesis of the alkyne-bearing DOPE carbamate m_A_-DOPE. Reagents and conditions: **(a)** *p*-nitrophenyl chloroformate, Et_3_N, THF, rt, 12 h, and 82%; **(b)** DOPE, 2:1 DCM/DMF, Et_3_N, 0°C to rt, 12 h, and 90%.

Fluoroazide **3** (Figure 1) was reacted with the linear alkyne functional group on the surface of m_A_-Lip 5 mol% m_A_-DOPE (Scheme 1) in 10 mM L_t_. Briefly, 500 μL of m_A_-Lip (1 equivalent, 0.25 µmol of m_A_-DOPE) was mixed with sodium ascorbate (2 equivalents, 0.5 µmol, 99 µL), CuSO_4_·5H_2_O (1 equivalent, 0.25 µmol, 76 µL), and an excess of fluoroazide **3** (2.5 equivalents, 0.625 µmol, 41 µL) in DMSO (5.8% v/v final DMSO concentration). The reaction vial was kept under orbital shaking at 40°C for 30 min and 1 h, respectively. The same procedure was performed for the CuAAC reaction on the surface of mA-Lip-MSLP-mApoE. After reaction completion, a small aliquot of the reaction mixture (100 µL) was collected for size, PDI, and ζ-potential measurements.

### 2.5 CyOctC reaction on the surface of m_CA_-Lip and m_CA_-Lip-MSLP-mApoE

**SCHEME 2:**
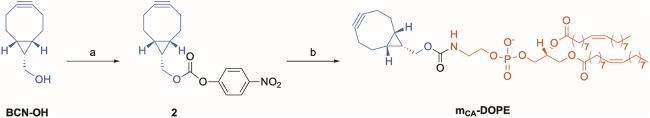
Synthesis of the cycloalkyne-bearing DOPE carbamate m_CA_-DOPE for copper-free click chemistry. Reagents and conditions: **(a)** *p*-nitrophenylchloroformate, pyridine, DCM, rt, 15 min, and 53%; **(b)** DOPE, 2:1 DCM/DMF, Et_3_N, 0°C to rt, 12 h, and 79%.

Fluoroazide **3** was reacted with the cyclic alkyne functional group on the surface of m_CA_-Lip 5 mol% m_CA_-DOPE (Scheme 2) in 10 mM Lt. Briefly, 500 µL of m_CA_-Lip (1 equivalent, 0.25 µmol of m_CA_-DOPE) was mixed with ultrapure water (175 µL) to ensure similar reagent concentrations and an excess of fluoroazide **3** (2.5 equivalents, 0.625 µmol, 41 µL) in DMSO (5.8% v/v final DMSO concentration). The reaction vial was kept under orbital shaking for 30 min at 50°C. The same procedure was performed for the CyOctC reaction on the surface of m_CA_-Lip-MSLP-mApoE. After reaction completion, a small aliquot of the reaction mixture (100 µL) was collected for size, PDI, and ζ-potential measurements.

### 2.6 Monitoring of the click reaction on the liposome surface and yield determination using TLC

An aliquot of the liposomal formulation (500 µL) was mixed with chloroform (2 mL) in a glass vial and vortexed. Then, the aqueous phase was removed, and the collected organic phase was concentrated and then solubilized with chloroform (200 µL). Before starting with the analysis, pre-coated TLC sheets (5 × 10 cm, 0.20 mm silica gel 60 ALUGRAM^®^ Xtra SIL/UV_254_, Macherey-Nagel, Germany) were conditioned with MeOH and heated using a heat gun. TLC sheets were allowed to run in a glass jar saturated with the mobile phase composed of a 75:25 (v/v) DCM: MeOH mixture. The click reactions were monitored using the following compounds as reference standards: cholesterol and sphingomyelin, mA-DOPE and mCA-DOPE (see Scheme 1, 2), m_A_-DOPE triazole (**4**) and m_CA_-DOPE (**5**) (see Figure 1); cholesterol and sphingomyelin were used as standard TLC spots. Cerium molybdate staining (Hanessian’s stain) was used to visualize the spots by dipping the TLC plate into the staining solution. After removing the excess staining solution, intense heating was applied to reveal the dark blue spots that appeared upon heating on a light blue background. Standards and sample solutions were manually spotted onto the TLC plates (Supplementary Figure S11).

### 2.7 Radiosynthesis

Solvents, reagents, and materials were purchased from Sigma-Aldrich and CARLO ERBA (Cornaredo, Italy). [^18^F]fluoride was produced using a cyclotron (Cyclone 18/9, IBA, Louvain la Neuve, Belgium) via the ^18^O (p,n)^18^F nuclear reaction by proton beam irradiation of a target containing 2 mL of >97% enriched [^18^O]water (Taiyo Nippon Sanso, Japan). Radiosynthesis procedures were performed using a commercially available automated system (Trasis AllinOne, Trasis, Ans, Belgium) located in a suitably shielded hot cell (MIP-2, Comecer, Castel Bolognese, Italy). Sep-Pak Light Waters Accell Plus QMA and Sep-Pak tC18 cartridges were purchased from Waters Corp. (Milford, United States). BabyBio Dsalt cartridges were purchased from Bio-Works (Uppsala, Sweden). Radiolabeled preparations and non-radiolabeled standards were analyzed by RP-HPLC on a JASCO PU-2089i System equipped with an automated injector, a DAD detector, and the Raytest Gabi Star radiochemical detector (JASCO, Cremella, Italy). Semi-preparative purification was performed using an RP-HPLC equipped with a Knauer WellChrom mod. K-2501 UV detector or using a Knauer P4.1S pump and a TOYDAD400 2Ch UV detector connected to the automated radiosynthesis system (Knauer, Berlin, Germany). The wavelength was set to 220 nm. The analytical RP-HPLC column (XTerra C18 250 × 4.6 mm, 5 μm) was purchased from Waters Corp., while the semi-preparative RP-HPLC column (Clarity Oligo-RP C18, 250 × 10 mm, 5 µm) was purchased from Phenomenex (Torrance, United States). Radio-TLC analyses were performed using a PerkinElmer Cyclone^®^ Plus, equipped with a Cyclone^®^ Plus Phosphor scanner and OptiQuant™ software.

#### 2.7.1 Radiosynthesis of 1-(azidomethyl)-4-4 ((2-[2-(2-[^18^F]fluoroethoxy) ethoxy] ethoxy) methyl)benzene (**[** ^**18**^ **F]3**)

**SCHEME 3:**
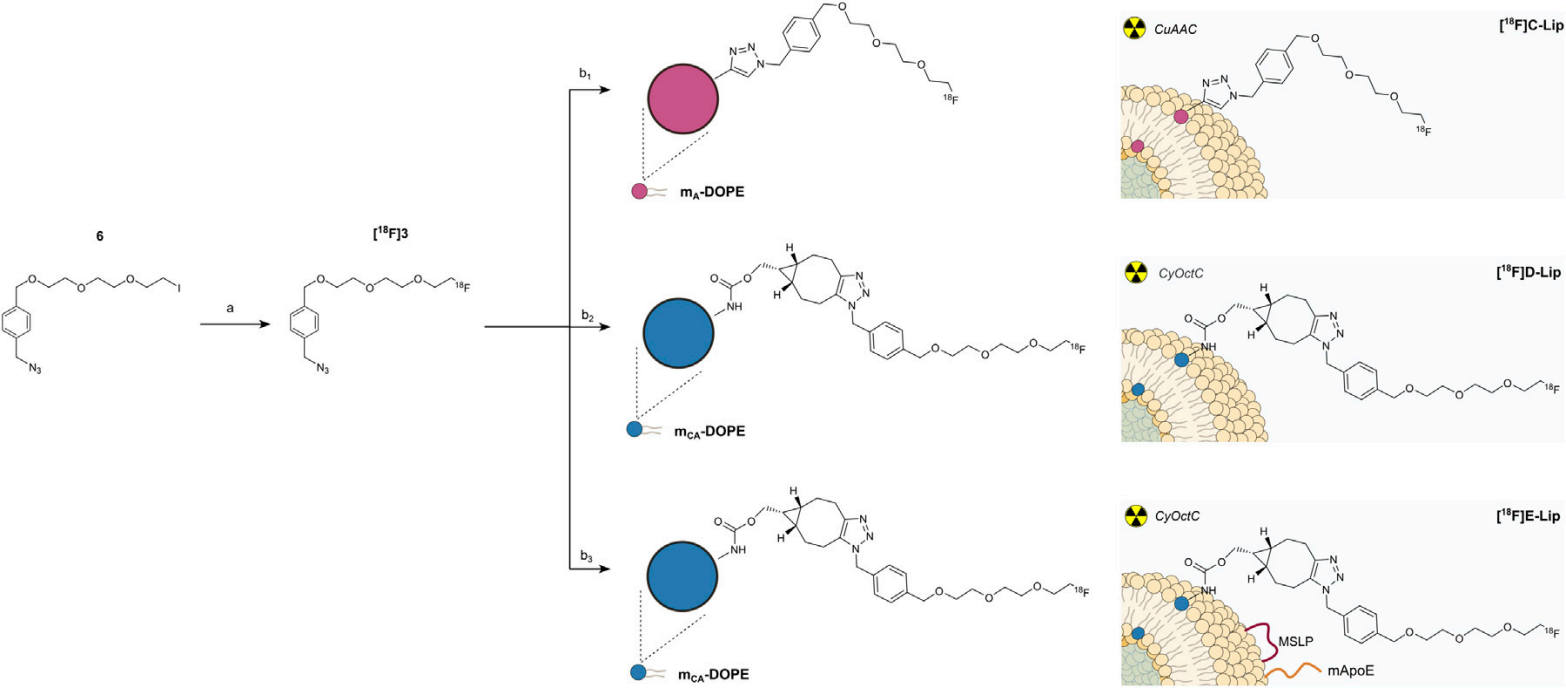
CuAAC and CyOctC protocols for liposome radiolabeling with fluorine-18. **(a)** [^18^F]KF, kryptofix 2.2.2, ACN, 20 min, 100°C, and ∼35%;**(b1)** m_A_-Lip, CuSO_4_·5H_2_O, ascorbic acid, water, 20 min, rt, ∼76%; **(b2)** m_CA_-Lip, water, 30 min, 40°C–50°C, and ∼40%; **(b3)** m_CA_-Lip-MSLP-mApoE, water, 30 min, 40°C–50°C, and ∼40%.

Approximately 40 GBq of cyclotron-produced [^18^F]fluoride solution was passed through a Sep-Pak Light QMA cartridge. A solution of potassium carbonate in water (2.51 mg, 0.018 mmol, 0.5 mL) was then passed through the cartridge, eluting the obtained K^+^[^18^F]^-^ directly in the reaction vial. A solution of Kryptofix 2.2.2 (15 mg, 0.04 mmol) in 1 mL acetonitrile (ACN) was added, and the solvent was azeotropically distilled at 100°C at reduced pressure in 12 min. Then, the reaction vial was cooled to 60°C, and the iodoazide **6** (see Scheme 3) solution (10 mg, 0.025 mmol) in anhydrous ACN (1 mL) was added. The reaction mixture was stirred for 20 min at 100°C. Then, the reaction vial was cooled down to 50°C. A 60:40 water: ACN solution (8 mL) was added, and the mixture was submitted to semi-preparative RP-HPLC. The collected fraction was diluted with water (30 mL) and passed through a tC18 Sep-Pak Plus cartridge previously conditioned with ethanol (10 mL) and water (10 mL). The cartridge was then washed with water (10 mL), and the final product **[** ^**18**^ **F]3** was eluted with ACN (0.8 mL) in the final container (radiochemical yield not corrected for decay (ndc): ∼35%, radiochemical purity >99%, molar activity: >1 TBq/µmol). The semi-preparative RP-HPLC conditions were as follows: clarity Oligo-RP 5 µm column, 250 × 10 mm; water: ACN gradient from 60:40 to 20:80 in 20 min, 5 mL min^−1^, 220 nm, UV detector. Rt: 17 min. The analytical RP-HPLC conditions were as follows: XTerra C18 5 µm column, 250 × 4.6 mm; water: ACN gradient from 60:40 to 20:80 in 20 min; 1 mL min^−1^, 220 nm, UV detector. Rt: 10.2 min (Supplementary Figure S13). Radio-TLC conditions were as follows: Xtra cartridge R_f_ **[** ^**18**^ **F]3** = 0.4 (water: ACN 90:10) (Supplementary Figure S14).

#### 2.7.2 CuAAC radiosynthesis on the surface of m_A_-Lip and formation of **[** ^**18**^ **F]C-Lip**

After purification, **[** ^**18**^ **F]3** was collected into the second automated system reactor, where ACN was removed by heating up to 85°C for 18 min. Then, the reactor was cooled to 30°C, and 500 µL of m_A_-Lip formulation (1.1× 10^13^ NP mL^−1^) and freshly prepared aqueous sodium ascorbate (99 μg, 0.5 µmol in 0.1 mL of water) were added, followed by aqueous CuSO_4_·5H_2_O (62.4 µg, 0.25 µmol in 0.1 mL). The reaction mixture was stirred for 20 min at rt. Next, the mixture (0.7 mL) was passed through two 5-mL prepacked BabyBio^TM^ Dsalt cartridges (10 mL total volume) connected in series, previously washed with water (50 mL), and followed by a nitrogen stream. Then, the cartridges were washed with water (7 mL, at a flow rate of 2 mL/min), and pure radiolabeled **[** ^**18**^ **F]C-Lip** was eluted in the first fraction (4 mL), which was collected in the final container to which the physiological saline solution (16 mL) was added (radiochemical yield not corrected for decay (ndc) = 9.6 ± 0.8%, radiochemical purity >95%, and total radiosynthesis time of 126 min), approximately 8 × 10^−4^ – 1 × 10^−3^ Bq/nanoparticle. Radio-TLC conditions were as follows: Xtra cartridge R_f_ **[** ^**18**^ **F]C-Lip** = 0 (water: ACN 90:10) (Supplementary Figures S15, S16).

#### 2.7.3 CyOctC radiosynthesis on the surface of m_CA_-Lip and m_CA_-Lip-MSLP-mApoE: formation of **[** ^**18**^ **F]D-Lip** and **[** ^**18**^ **F]E-Lip**

**[** ^**18**^ **F]3** was synthesized as described above and collected in the second automated system reactor. ACN was removed by heating up to 85°C for 18 min, and then the reactor was cooled down to 30°C. Either an m_CA_-Lip formulation or an m_CA_-Lip-MSLP-ApoE formulation (500 μL, 1.1 × 10^13^ NP mL^−1^) was added, and the reaction mixture was heated at 40°C–50°C for 30 min. The mixture (0.5 mL) was passed through two BabyBio^TM^ Dsalt cartridges (10 mL of total volume) connected in series, previously washed with water (50 mL) under a nitrogen stream. Then, water (7 mL) was pumped into the cartridges (flow 2 mL/min), and either pure radiolabeled **[** ^**18**^ **F]D-Lip** or **[** ^**18**^ **F]E-Lip** was eluted in the first fraction (4 mL) and then collected in the final container to which the physiological saline solution (16 mL) was added. **[** ^**18**^ **F]D-Lip**: RCYndc = 5.6 ± 0.9%, purity >95%, and total radiosynthesis time of 129 min **[** ^**18**^ **F]E-Lip**: RCYndc = 4.2 ± 1.0%, purity >95%, and total radiosynthesis time of 129 min ca 2 × 10^−4^ – 6 × 10^−4^ Bq/nanoparticle. Radio-TLC conditions were as follows: Xtra cartridge R_f_ **[** ^**18**^ **F]D, E-Lip** = 0 (water: ACN 90:10) (Supplementary Figures S17–19).

#### 2.7.4 Liposome disruption procedure

Approximately 10 MBq (50 µL) of **[** ^**18**^ **F]C, D,** and **E-Lip** formulations were added to 250 µL of chloroform and vortexed. Then, 250 µL of ethanol was added. The mixture was further vortexed, and 2 µL aliquots were deposited on the TLC strip. R_f_ **[** ^**18**^ **F] 4,5** (Figure 1) = 0.8 (DCM:MeOH 75:25) (Supplementary Figures S20–22).

### 2.8 Cell cultures

Orthotopic glioma mouse model (Gli36ΔEGFR) cells were cultured in Dulbecco’s modified Eagle medium (DMEM) supplemented with 10% heat-inactivated fetal bovine serum (FBS), 2 mM L-glutamine, and 50 IU mL^−1^ penicillin/streptomycin (P/S) (Euroclone, United Kingdom) and maintained in a 5% CO_2_/95% air atmosphere at 37°C. Careful attention was given to the cell culture to check for typical growth patterns and phenotype.

### 2.9 Cell viability assay on hCMEC/D3 cells

The effect of **[** ^**18**^ **F]C-Lip**, after complete fluorine-18 decay (48 h after radiosynthesis), on hCMEC/D3 cell viability was assessed by the MTT assay (Stewart, 1980). hCMEC/D3 cells were seeded in a 96-well plate at a density of 20,000 cells per well. Liposome concentrations ranging from 1.12 × 10^10^ to 4.48 × 10^11^ NP/mL were added to the culture medium. After 24 h, the assay was performed as per the manufacturer’s protocol, and the absorbance was measured at 690 and 570 nm using a microplate reader (SPECTROstar Nano, BMG LABTECH, Ortenberg, Germany). Results are presented as the mean of three independent experiments ±SD, considering untreated cells as 100% of cell viability. Data were analyzed using GraphPad Prism 8 software, and the statistical analysis was performed using two-way ANOVA.

### 2.10 Animal model and study design

The institutional guidelines for the care and use of experimental animals, approved by the Italian Ministry of Health (n° 1174/2020-PR), were followed for the animal experiments. Mice were housed in a controlled environment with food and water available *ad libitum*, under a regular light/dark cycle, constant temperature (23°C), and relative humidity (60%–70%). The total body biodistribution of **[** ^**18**^ **F]C-Lip**, **[** ^**18**^ **F]D-Lip**, and **[** ^**18**^ **F]E-Lip** was evaluated *in vivo* by PET/CT on Gli36ΔEGFR cells at tumor onset. Female nude mice, 7–8 weeks old (Envigo RMS SRL., S. Pietro al Natisone, Italy), were intracranially injected with 5 × 10^5^ Gli36ΔEGFR cells, as previously described (Valtorta et al., 2021). Animal health status was monitored daily after cell injection to observe changes in body weight or clinical signs of disease, such as fur, eye, and motor impairment. Ten days after surgery, a T2-weighted magnetic resonance imaging (MRI) was performed on mice using a 7T small animal magnetic resonance scanner (Bruker, BioSpec 70/30 USR, Paravision 5.1, Germany). Two days before the biodistribution study, a PET scan with the proliferation radiopharmaceutical [^18^F]FLT was performed on the mice to confirm the tumor growth.

### 2.11 *In vivo* PET/CT study

First, we evaluated the *in vivo* biodistribution of **[** ^**18**^ **F]C-Lip** (n = 4). Subsequently, a separate group of animals was included in the study (n = 4) to evaluate the *in vivo* biodistribution of **[** ^**18**^ **F]D-Lip** and **[** ^**18**^ **F]E-Lip**, performing PET acquisition on different but consecutive days. Mice were injected with 3.8 ± 0.1 MBq of **[** ^**18**^ **F]C-Lip**, 4.0 ± 0.2 MBq of **[** ^**18**^ **F]D-Lip**, and 3.8 ± 0.1 MBq of **[** ^**18**^ **F]E-Lip** via the tail vein. Whole-body PET/CT scans of the mice were obtained using a small-animal PET/CT scanner, ß-X-CUBE (Molecubes, Ghent, Belgium), at 10, 60, 120, and 180 min after radiotracer injection. Each scan lasted 30 min while the mice were maintained under anesthesia (4% isoflurane during induction and 2% during maintenance in air). In both cases, PET-[^18^F]FLT and T2w MRI imaging studies were conducted as previously described (Valtorta et al., 2021).

### 2.12 Image quantification

PMOD 4.105 software (Zurich, Switzerland) was used for quantification purposes. First, PET/CT brain images were co-registered with MRI images, and two volumes of interest (VOIs) were defined. The first VOI, with a volume of 4 mm^3^, was a control VOI that covered the healthy left striatum (contralateral, Cl). This control VOI was drawn on the axial MR images, adjusted in the other imaging planes, and then copied onto the PET images of each mouse. The second VOI was located in the tumor-affected brain hemisphere and centered on the mouse lesion. VOIs were also drawn on peripheral organs (lung, kidney, heart, liver, muscle, bone, spleen, and intestine). Data were expressed as SUVmean, calculated as a percentage of the injected dose normalized to mouse mass after correction for the physical decay of fluorine-18 and the tumor-to-contralateral (T/Cl) ratio.

### 2.13 Statistical analysis

Data were reported as mean ± SD. For statistical analysis, two-way ANOVA Bonferroni’s multiple comparison test was performed using Prism 9 (GraphPad Software Inc., CA, United States). Differences were considered statistically significant when p < 0.05. The area under the curve (AUC) was also computed using the Prism 9 software.

## 3 Results

### 3.1 Chemical synthesis of key reagents

#### 3.1.1 Synthesis of alkyne-functionalized DOPE constructs

DOPE was modified with two alkyne-containing constructs (here, defined as modified DOPE or m-DOPE), and their synthetic procedures were rationally designed to allow their radiolabeling on the liposome surface. In detail, having extensively studied the coupling conditions between DOPE and various alkyne-containing derivatives, we activated propargyl alcohol by reaction with *p*-nitrophenyl chloroformate (step a, Scheme 1), yielding carbonate **1**. Conjugation of the latter with DOPE under standard, mild conditions resulted in the target carbamate construct m_A_-DOPE (step b, Scheme 1) with a good yield and purity. The NMR spectra of **1** (Supplementary Figures S1, S2) and m_A_-DOPE (Supplementary Figures S5, S6) and the HRMS spectrum of m_A_-DOPE (Supplementary Figure S9) are reported in the Supporting Information.

Moreover, we targeted a similar m-DOPE construct that would not require copper(I) for its bio-orthogonal “click” reaction on multi-functionalized liposomes. The commercially available cyclooctyne alcohol BCN-OH was selected since its triple bond undergoes a bio-orthogonal click reaction in a non-regioselective, copper(I)-free environment (Blenke et al., 2015). The preparation of the target cyclooctyne carbamate construct m_CA_-DOPE required the same carbonate activation to obtain the key carbonate intermediate **2** (step a, Scheme 2), followed by conjugation with DOPE (step b, Scheme 2), which yielded pure m_CA_-DOPE in a slightly lower but satisfactory yield. The NMR spectra of **2** (Supplementary Figures S3, S4) and m_CA_-DOPE (Supplementary Figures S7, S8) and the HRMS spectrum of m_CA_-DOPE (Supplementary Figure S10) are reported in the Supporting Information.

#### 3.1.2 Synthesis of unlabeled [^19^F]fluoroazide (**3**), radiolabeled **[** ^**18**^ **F]**fluoroazide **([** ^**18**^ **F]3)**, and **[** ^**19**^ **F]**DOPE-triazole standards (**4**,**5**)

The two key fluoroazides **3** and **[** ^**18**^ **F]3** (Figure 1) were prepared as recently reported (Lugato et al., 2017). Cycloaddition reactions between [^19^F]fluoroazide and m_A_-DOPE (CuAAC) and m_CA_-DOPE (“copper-free” cycloaddition) were successfully performed, yielding compounds **4** and **5**, respectively. The detailed radiosynthetic procedures are reported in the Experimental Section.

### 3.2 Liposome functionalization

#### 3.2.1 Liposome functionalization with m_A_-DOPE and conjugation with non-radioactive fluoroazide (**3**) via CuAAC

Liposomes were initially prepared to assess a suitable formulation protocol for the CuAAC radiolabeling reaction. First, the optimal molar percentage of m-DOPE constructs to be used in liposomal formulations was assessed using m_A_-DOPE. Liposomes containing different concentrations of m_A_-DOPE (m_A_-Lip) were prepared, and hydrodynamic size, PDI, and surface charge (ζ-pot) were measured (Table 1). All m_A_-Lip were smaller than 150 nm, showing low to medium dispersity and negative ζ-pot. As expected, the liposomal Z potential increased in absolute value as a function of m_A_-DOPE concentration. Particle size was measured by DLS and NTA, the latter showing an average size of ∼120 nm for all formulations. Accordingly, PDI (DLS) and Span (NTA) values were ≥0.1 and 1, respectively, indicating a low to medium dispersity of the liposomes (Blenke et al., 2015; Ribeiro et al., 2018; Danaei et al., 2018).

**TABLE 1:** Physicochemical properties of m_A_-Lip preparations at different m_A_-DOPE concentrations.

	mA-DOPE^a^	D_h_ ^b^	PDI	ζ-pot^c^	D_h_ ^d^	[NP]^e^	Span^f^	Lipid yielding^g^
m_A_-Lip 0	0	138.8 ± 1	0.1	−13 ± 1	122 ± 1	1.8	0.60	50
m_A_-Lip 1	1	130.5 ± 2	0.07	−21 ± 3	119 ± 1	0.5	0.62	56
m_A_-Lip 2	2	134.2 ± 2	0.07	−27 ± 1	121 ± 1	1.0	0.51	54
m_A_-Lip 5	5	120.6 ± 1	0.1	−42 ± 2	122 ± 2	1.1	0.63	42
m_A_-Lip 10	10	139.3 ± 3	0.2	−45 ± 2	121 ± 1	2.3	0.65	50

^a^ [%mol].

^b^ [nm] ± SD obtained by DLS.

^c^ [mV] ± SD.

^d^ size obtained from NTA, [nm] ± SD.

^e^ ×10^13^ NP mL^-1^.

^f^ calculated from NTA D90, D50, and D10 parameters.

^g^ calculated using the Stewart Assay.

Subsequently, m_A_-Lip with a 5% mol m_A_-DOPE content was selected for further CuAAC reaction. The CuAAC reaction was performed by incubating 5% mol m_A_-Lip with CuSO_4_·5H_2_O, ascorbic acid, and fluoroazide **3**. By TLC monitoring, the anchoring of compound **3** by triazole linkage to the liposome surface was confirmed after 30 min of reaction at room temperature. Considering the different retention factors (R_f_) between unreacted m_A_-DOPE (R_f_ = 0.62–0.65) and the CuAAC fluorotriazole DOPE (product **4**, Figure 1) (Rf = 0.71–0.77), we estimated the reaction yield (Supplementary Figure S11) to be ∼75 ± 11%. Total conversion of m_A_-DOPE to **4** could not be achieved due to the known m_A_-DOPE fraction orienting its alkyne group toward the internal membrane of the liposomes (Blenke et al., 2015). Liposome size, PDI, and Z potential did not significantly change after the CuAAC reaction (Table 2).

**TABLE 2:** Physicochemical characterization of 5% mol mA-Lip formulations after incubation with fluoroazide **3** under CuAAC conditions.

Time, min	D_h_ ^a^	PDI	ζ-pot^b^
0	125 ± 2	0.1	−44 ± 1
30	135 ± 1	0.1	−41 ± 2
60	133 ± 1	0.07	−43 ± 1

^a^ [nm] ± SD.

^b^ [mV] ± SD.

Therefore, the same experiment was performed on liposomes containing 5% mol m_A_-DOPE and functionalized with the MSLP or mApoE peptide (Giofrè et al., 2022). The results showed that the “click” reaction on liposomes functionalized with MSLP, i.e., m_A_-Lip-MSLP, did not affect the physicochemical parameters of the nanoparticles. On the contrary, the functionalization of liposomes with mApoE, both alone (m_A_-Lip-mApoE) or in combination with MSLP (m_A_-Lip-MSLP-mApoE), induced an increase in size and a Z potential closer to neutrality, indicating a possible destabilization of the liposomal formulation. The incubation of m_A_-Lip-MSLP-mApoE with sodium ascorbate or CuSO_4_ induced a slight size increase to 176 ± 2 nm and 202 ± 2 nm, respectively (Zhang et al., 2022).

In order to avoid the use of sodium ascorbate and CuSO_4_, m_A_-DOPE was replaced with a cyclic alkyne (m_CA_-DOPE) as its use as a “click” reaction reagent does not need to be copper-catalyzed, taking advantage of the free energy intrinsic to the “strained” structure of the cyclooctyne.

#### 3.2.2 Liposome functionalization with m_CA_-DOPE and labeling with fluoroazide **3** via copper-free CyOctC

The CyOctC protocol entailed the reaction between m_CA_-DOPE and fluoroazide **3**. DMSO was required to solubilize the fluoroazide in a manual, non-automated reaction protocol. The substitution of m_A_-DOPE with m_CA_-DOPE (Table 3) did not affect the physicochemical features of the liposomes. As expected, a slight increase in size and PDI was detected with m_CA_-Lip-MSLP only and dually functionalized liposomes with mApoE and MSLP.

**TABLE 3:** Physicochemical properties of m_CA_-Lip formulations.

	D_h_ ^a^	PDI	ζ-pot^b^
m_CA_-Lip	119 ± 1	0.122	−36 ± 1
m_CA_-Lip-MSLP	140 ± 1	0.228	−24.2 ± 2
m_CA_-Lip-MSLP-mApoE	145.2 ± 2	0.203	−27 ± 2

^a^ [nm] ± SD.

^b^ [mV] ± SD.

Then, m_CA_-Lip-MSLP-mApoE was reacted with fluoroazide **3** and dissolved in DMSO, according to a CyOctC protocol. The resulting fluorine-19-labeled m_CA_-Lip-MSLP-mApoE (**[** ^**19**^ **F]E-Lip** Scheme 3) was characterized by 207 ± 1 nm diameter, PDI <0.2, and 15.5 ± 2 mV ζ -potential.

### 3.3 Radiolabeling of liposomes via CuAAC and copper-free CyOctC reactions

Liposome surface radiolabeling with **[** ^**18**^ **F]3** was performed on an automated radiosynthesis system, AllinOne (Trasis, Belgium). The radiolabeling procedure entailed two radiosynthetic steps: 1) the synthesis of purified **[** ^**18**^ **F]3** and 2) a “click” cycloaddition reaction (CuAAC or CyOctC) between the radiolabeled azide and an m_A_-Lip or m_CA_-Lip on the liposome surface. The radiosynthetic procedures were performed by modifying a “general purpose” automated method for fluorine-18 introduction on biologically active molecules under mild conditions using the CuAAC approach, previously developed in our laboratory (Iannone et al., 2022).

#### 3.3.1 Radiosynthesis of **[** ^**18**^ **F]3**

Scheme 3 shows the SN2 nucleophilic substitution between [^18^F]F^−^ and iodoazide precursor **6** (step a). Heating in ACN (≈100°C) was mandatory to accomplish optimal conversion to **[** ^**18**^ **F]3**, which was then purified from byproducts of the S_N_2 reaction by semi-preparative RP-HPLC in order to obtain the highest radiochemical purity (>99%) and molar activity (>1 TBq/µmol) before attempting either CuAAC (step b1) or CyOctC (step b2) on the alkyne residues located on the liposome surface of m_A_-Lip and m_CA_-Lip, respectively. Chemical purity was also satisfactory, as only impurities in trace amounts were detected at 220 nm, which is the wavelength of maximum absorption for the compounds of interest (Supplementary Figures S13, S14) (Lugato et al., 2017).

#### 3.3.2 Liposome surface radiolabeling with the CuAAC reaction (step b1)

Purified **[** ^**18**^ **F]3** and m_A_-DOPE liposomes were mixed with ascorbic acid and copper sulfate pentahydrate. The CuAAC cycloaddition was carried out at room temperature in an aqueous solution for 20 min under a nitrogen stream. Radio-TLC monitoring of the reaction showed an average ∼76% conversion of **[** ^**18**^ **F]3** to **[** ^**18**^ **F]C-Lip** (Supplementary Figure S15). The chemical structure of **[** ^**18**^ **F]C-Lip** is reported in Scheme 3. The CuAAC reaction was performed both with and without DMSO addition. No differences in terms of radiochemical conversion, purity, time, and temperature were noted.

Purification of the crude CuAAC mixture, containing the desired **[** ^**18**^ **F]C-Lip**, unreacted **[** ^**18**^ **F]3**, and degradation by-products, was accomplished using size-exclusion chromatography (SEC). In this regard, Bio-Works^®^ prepacked BabyBio^TM^ D salt cartridges of different volumes were tested, and the best **[** ^**18**^ **F]C-Lip** purification conditions were achieved by connecting two of the abovementioned cartridges (10 mL of total elution volume) in series.

A radiochemical yield of 9.6% ± 0.8%, calculated from the initial amount of fluorine-18, and radiochemical purity of >95% (Supplementary Figure S16) were obtained, which are suitable for further use in preclinical testing *in vivo*; the total radiosynthesis time was approximately 126 min (n = 4), with molar activity in the range of 8 × 10^−4^ – 1 × 10^−3^ Bq/nanoparticle. The entire radiosynthetic process was automated using a Trasis AllinOne Automated system (Supplementary Figure S23). Conversely, smaller cartridge volumes could not properly separate radiolabeled liposomes from radioactive byproducts (Supplementary Figures S15, S16). The BabyBio^TM^ Dsalt cartridges were then eluted with water, and a physiological saline solution was added to the collected fraction containing pure **[** ^**18**^ **F]C-Lip**, obtaining a final fluorine-18 radiolabeled liposome solution ready for injection (size <200 nm, PDI <0.2, and charge −25.3 ± 3.5 mV).

In addition, the effect of **[** ^**18**^ **F]C-Lip** on cell viability was measured *in vitro* after complete fluorine-18 decay (approximately 48 h after radiosynthesis) using immortalized human endothelial cells (hCMEC/D3); the results showed that liposomes did not affect cell viability, which remained comparable to that of untreated cells (Supplementary Figure S25).

#### 3.3.3 Liposome radiolabeling with the copper-free CyOctC reaction (step b2)

A different method, involving copper-free CyOctC “click” radiosynthesis, was developed, leveraging the improved reactivity of “strained” cycloalkynes. This approach enables a [3 + 2] cycloaddition reaction between the surface-exposed alkyne groups of cyclooctyne-containing m_CA_-DOPE and **[** ^**18**^ **F]3** without the need for metal catalysts (step b2, Scheme 3). The radiolabeling reaction was initially performed by heating at 50°C for 30 min, and a moderate but still acceptable ∼40% cycloaddition/conversion of **[** ^**18**^ **F]3** into the final **[** ^**18**^ **F]D-Lip** was obtained (Scheme 3). Considering that a reaction temperature of approximately 50°C might not be ideal for the stability of biological molecules, attempts to carry out the radiolabeling reaction by decreasing the temperature to 40°C were then performed, with comparable results regarding radiochemical yield and purity (Supplementary Figure S18).

The purification procedure, using size-exclusion chromatography (Bio-Works^®^ prepacked BabyBio^TM^ Dsalt cartridges), was the same as described above for the CuAAC protocol. The physicochemical parameters of **[** ^**18**^ **F]D-Lip** after radiolabeling were compared to those from a CuAAC protocol: size <200 nm, PDI <0.2, and charge < −30 mV. Finally, the optimized CyOctC protocol conditions were applied to the radiolabeling of the desired functionalized **[** ^**18**^ **F]E-Lip**, which was obtained with a radiochemical yield (not decay-corrected) of 4.2% ± 1.0% and a radiochemical purity >95% (Supplementary Figure S19), with a total radiosynthesis time of 129 min (n = 5) (size 210 ± 1.1 nm, PDI <0.2, and charge −20.5 ± 2.0); the molecular activity was approximately 2 × 10^−4^ – 6 × 10^−4^ Bq/nanoparticle. As expected, despite functionalization, an increase in nanoparticle diameter was not observed (see discussion in Section 4.1). Starting from an initial amount of ∼100 GBq of fluorine-18, it was possible to obtain 4–10 GBq of radiolabeled liposome formulation, representing an appropriate radioactivity amount for subsequent preclinical (and potentially clinical) applications.

### 3.4 *In vivo* PET/CT study

Mice orthotopically inoculated with Gli36ΔEGFR cell lines presented brain hyper-intense lesions on T2w MRI and showed uptake of [^18^F]FLT scans. After tumor onset, brain and systemic biodistribution of **[** ^**18**^ **F]C-Lip**, **[** ^**18**^ **F]D-Lip**, and **[** ^**18**^ **F]E-Lip** formulations were evaluated by PET/CT imaging up to 3 hours post-injection (n = 3). This time frame was chosen based on previous results, showing no significant changes in this time frame in the amount of mApoE-MSLP-liposomes reaching the brain after a single systemic administration in mice (Bana et al., 2014; Taiarol et al., 2022). The results of the *in vivo* peripheral distribution (i.e., liver, heart, lungs, kidneys, muscle, small intestine, spleen, and bone) are summarized in Table 4 (expressed as SUV mean values). PET image quantification data of non-functionalized **[** ^**18**^ **F]C-Lip** and **[** ^**18**^ **F]D-Lip** showed higher uptake and a slower washout over time in the majority of peripheral organs than those on **[** ^**18**^ **F]E-Lip** (Table 4). In particular, in the liver, spleen, and intestine, the initial radioactivity accumulation of **[** ^**18**^ **F]D-Lip** was significantly higher than that of **[** ^**18**^ **F]E-Lip**. This effect was maintained over time, as indicated particularly in the liver and intestine. In addition, **[** ^**18**^ **F]D-Lip** showed higher uptake in the liver, spleen, and small intestine than **[** ^**18**^ **F]C-Lip**, indicating a different effect on nanoparticle distribution exerted by m_CA_-DOPE and m_A_-DOPE-fluorinated residues. In other organs, radioactivity accumulation was lower, with no evidence of time-dependent accumulation. Overall, these results indicate that mApoE influences nanoparticle accumulation, showing lower levels in the reticular endothelial organ and intestine than non-functionalized **[** ^**18**^ **F]D-Lip** particles. All nanoparticles displayed low levels of radioactivity concentration in bone, with no time-dependent increase, suggesting negligible levels of *in vivo* defluorination, particularly for **[** ^**18**^ **F]C-Lip**. The higher uptake observed for **[** ^**18**^ **F]D-Lip** and **[** ^**18**^ **F]E-Lip** than for **[** ^**18**^ **F]C-Lip** may also be explained by a different homing of **[** ^**18**^ **F]D-Lip** and **[** ^**18**^ **F]E-Lip** in bone marrow cells compared to that of **[** ^**18**^ **F]C-Lip** (Lian et al., 2024).

**TABLE 4:** Data reported for the peripheral *in vivo* biodistribution analysis of **[** ^**18**^ **F]C-Lip**, **[** ^**18**^ **F]D-Lip**, and **[** ^**18**^ **F]E-Lip** derivatives. Data are reported as the SUV mean of each volume of interest.

Tissue SUV mean	[^18^F]C-Lip, (n = 3)				[^18^F]D-Lip, (n = 3)				[^18^F]E-Lip, (n = 3)			
Time, min	10	60	120	180	10	60	120	180	10	60	120	180
Lungs	0.65 ± 0.1	0.3 ± 0.05	0.4 ± 0.07	0.28 ± 0.04	0.77 ± 0.08	0.4 ± 0.09	0.35 ± 0.11	0.51 ± 0.04	0.35 ± 0.06	0.31 ± 0.04	0.29 ± 0.05	0.26 ± 0.04
Kidneys	0.59 ± 0.2	0.27 ± 0.04	0.38 ± 0.2	0.38 ± 0.09	0.94 ± 0.26	0.5 ± 0.11	0.43 ± 0.17	0.62 ± 0.21	0.34 ± 0.12	0.33 ± 0.16	0.42 ± 0.22	0.18 ± 0.09
Heart	0.56 ± 0.1	0.3 ± 0.07	0.38 ± 0.04	0.25 ± 0.05	1 ± 0.16	0.54 ± 0.06	0.44 ± 0.07	0.76 ± 0.13	0.39 ± 0.06	0.33 ± 0.02	0.27 ± 0.004	0.2 ± 0.5
Liver	5.28 ± 1.3**	2.19 ± 0.5****	2.15 ± 0.4*	1.35 ± 0.21****	6.67 ± 0.5****	3.76 ± 0.3****	3.03 ± 0.6*	4.87 ± 0.75****	3.08 ± 0.7****	2.64 ± 0.44	2.17 ± 0.66	2.16 ± 0.5
Muscle	0.13 ± 0.03	0.15 ± 0.02	0.17 ± 0.05	0.18 ± 0.02	0.26 ± 0.08	0.26 ± 0.07	0.22 ± 0.08	0.33 ± 0.04	0.1 ± 0.02	0.11 ± 0.01	0.09 ± 0.02	0.1 ± 0.02
Bone	0.19 ± 0.1	0.19 ± 0.05	0.27 ± 0.1	0.2 ± 0.09**	0.94 ± 0.28	1.23 ± 0.2***	0.88 ± 0.15	1.62 ± 0.66	0.7 ± 0.09	1.01 ± 0.2**	0.99 ± 0.05	1.02 ± 0.21
Spleen	1.46 ± 0.4****	1.06 ± 0.3**	0.19 ± 0.51	1.05 ± 0.52*	3.69 ± 1.7****	1.93 ± 0.96	1.44 ± 0.48	2.18 ± 0.75	1.62 ± 0.28	1.62 ± 0.26	1.59 ± 0.35	1.46 ± 0.3
Intestine	2.21 ± 0.2****	1.81 ± 0.2****	3.68 ± 1.1*	2.29 ± 0.82****	5.31 ± 0.4****	5.31 ± 0.2****	4.62 ± 0.8*	4.06 ± 1.58****	2.08 ± 0.17	2.81 ± 0.1***	3.65 ± 0.57	1.46 ± 0.23
	*p, **p, ***p, ****p vs. [^18^F]D-Lip				*p, **p, ***p, ****p vs. [^18^F]E-Lip				*p, **p, ***p, ****p vs. [^18^F]C-Lip			

Average values ±SD were calculated per group at 10, 60, 120, and 180 min. Significance levels are indicated as follows:*, p < 0.05; **, p < 0.01; ***, p < 0.001; and ****, p < 0.0001 by two-way ANOVA Bonferroni’s multiple comparison test.

**FIGURE 2:**
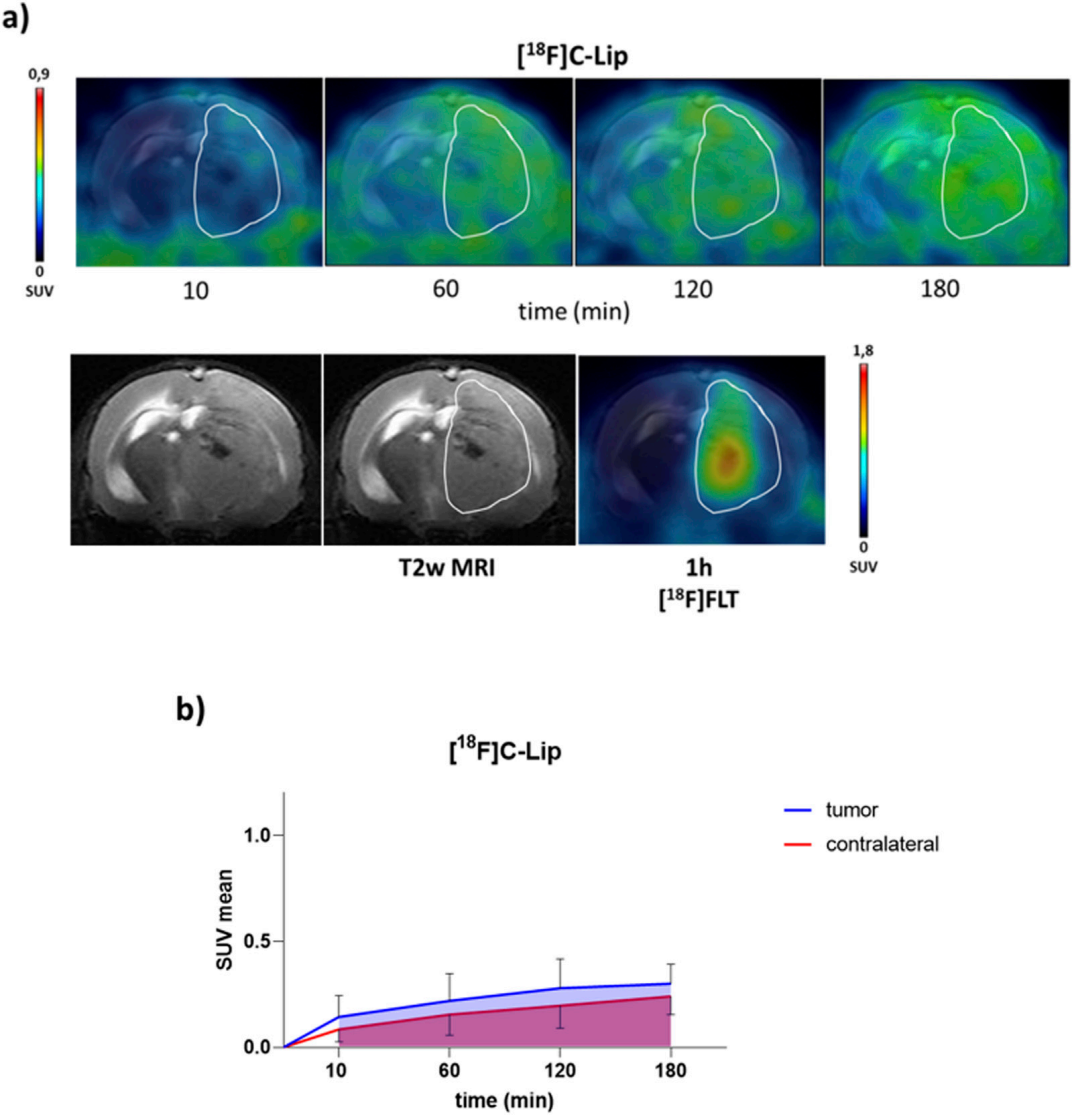
PET/CT brain uptake evaluation of [^18^F]C-Lip in a GBM orthotopic model (Gli36ΔEGFR cells). Gli36ΔEGFR tumor-bearing nude mice were i.v. injected with **[** ^**18**^ **F]C-Lip** (∼3.8 MBq/mouse). Radiotracer uptake was assessed after 10, 60, 120, and 180 min post-injection by whole-body PET/CT acquisitions. **(a)** Representative T2w MRI image (transaxial) of the brain (bottom left) fused with PET images of [^18^F]FLT (bottom right) and **[** ^**18**^ **F]C-Lip** (upper) from the same mouse for each experimental condition (the scale bar is reported as the SUV mean). The white line indicates the tumor area as depicted in the MRI images (T2w) and transferred to the PET images. [^18^F]FLT PET imaging was used as a clinical standard radiotracer to detect GBM tumor tissue. **(b)** The uptake quantification results are expressed as the standard uptake value mean (SUV mean). Curves, mean ± SD (n = 3 mice).

**FIGURE 3:**
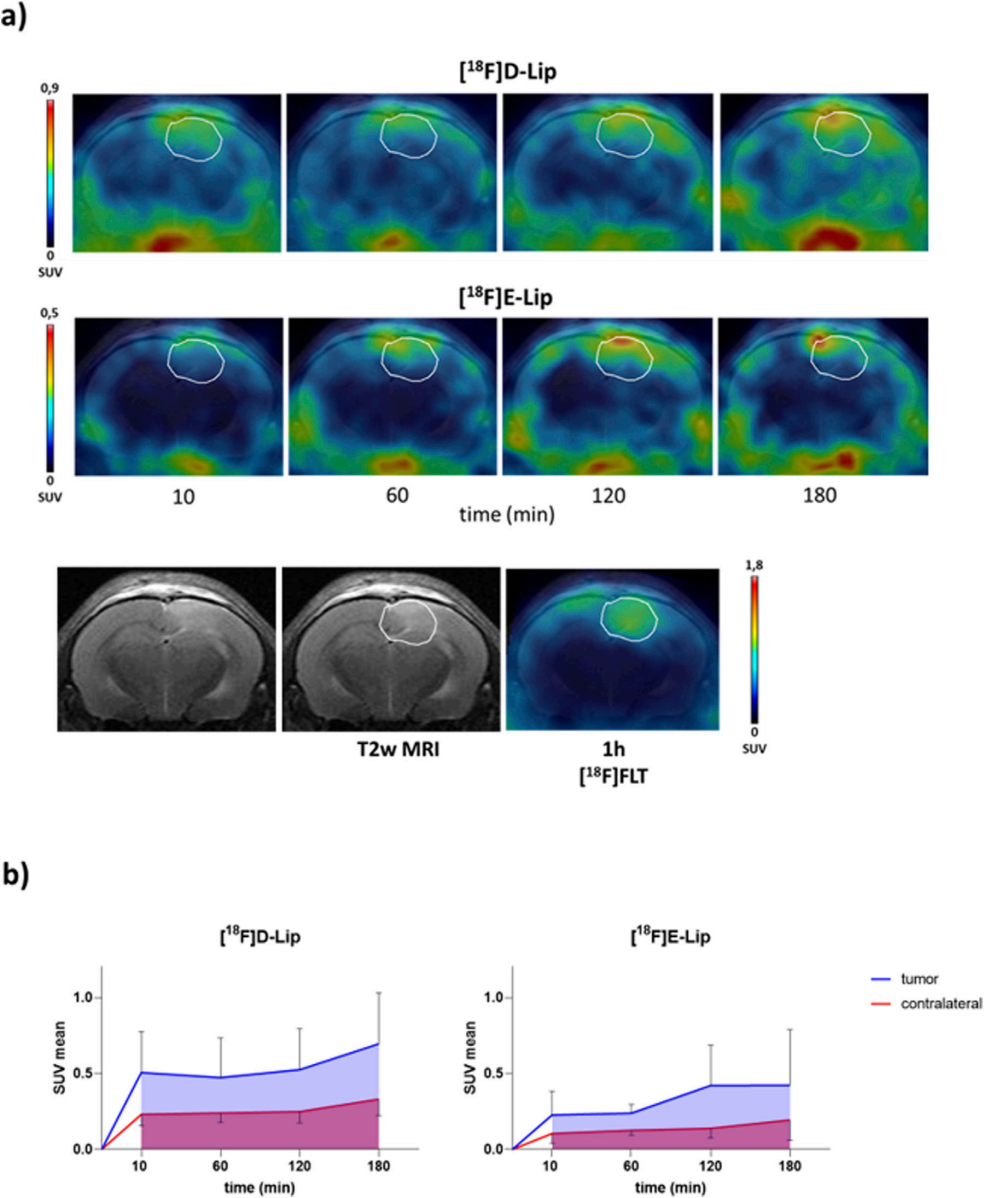
PET/CT brain uptake evaluation of [^18^F]D-Lip and [^18^F]E-Lip in a GBM orthotopic model (Gli36ΔEGFR cells). Gli36ΔEGFRitumor-bearing nude mice were i.v. injected with either **[** ^**18**^ **F]D-Lip** or **[** ^**18**^ **F]E-Lip** (∼3.8 MBq/mouse). Radiotracer uptake was assessed after 10, 60, 120, and 180 min post-injection by whole-body PET/CT acquisitions. **(a)** Representative T2w MRI image (transaxial) of the brain (bottom left) fused with PET images of [^18^F]FLT (bottom right), **[** ^**18**^ **F]D-Lip** (upper), and **[** ^**18**^ **F]E-Lip** (middle) from the same mouse for each experimental condition (the scale bar is reported as the SUV mean). The white line indicates the tumor area, as depicted in the MRI images (T2w) and transferred to the PET images. [^18^F]FLT PET imaging was used as a clinical standard radiotracer to detect GBM tumor tissue. **(b)** The uptake quantification results are expressed as the standard uptake value mean (SUV mean). Curves, mean ± SD (n = 3 mice).

All radiolabeled liposomes preferentially accumulated in the brain tumor over time, compared to the contralateral healthy parenchyma (Figures 2a, 3a), with similar tumor uptake values for **[** ^**18**^ **F]D-Lip** and **[** ^**18**^ **F]E-Lip** and lower for **[** ^**18**^ **F]C-Lip** (0.52 ± 0.27, 0.42 ± 0.26, and 0.28 ± 0.14 SUV mean at 120 min, respectively) (Figures 2b, 3b). Between tumor and normal brain parenchyma, no significant differences in the AUC values were detected for all formulations (AUC of SUV values, tumor vs. contralateral brain: 0.721 vs. 0.513 for **[** ^**18**^ **F]C-Lip**; 1.6 vs. 0.768 for **[** ^**18**^ **F]D-Lip;** and 0.984 vs. 0.413 for **[** ^**18**^ **F]E-Lip**) (Figures 2b, 3b).

## 4 Discussion

**SCHEME 4:**
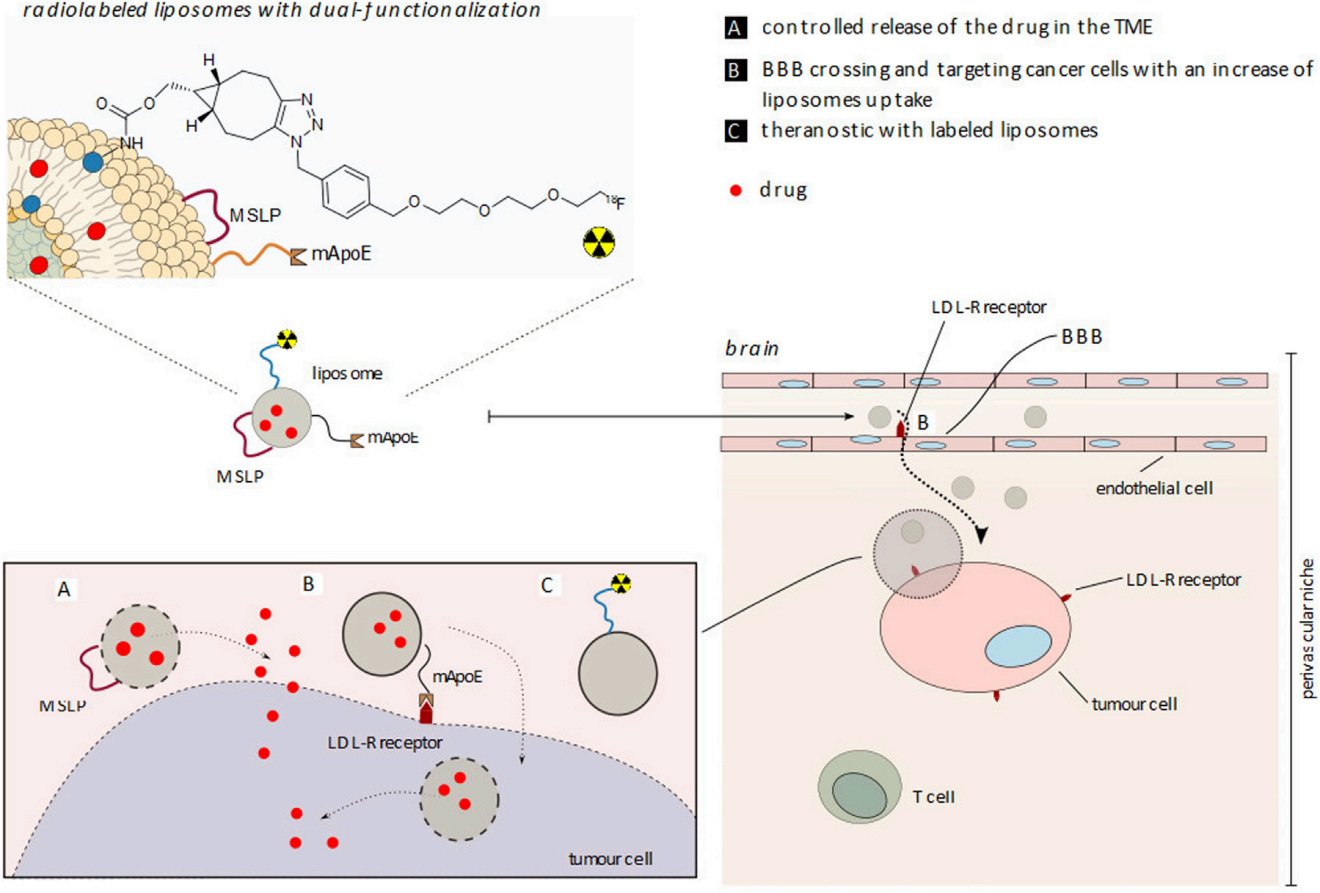
Representation of **[** ^**18**^ **F]E-Lip** liposome functionalization with apolipoprotein E (mApoE) and metalloproteinase (MMP) sensitive lipopeptide (MSLP) for cancer cell uptake and drug release in a therapy, diagnostic and future theranostic approach.** (A)** Controlled release of the drug in TME; **(B)** BBB crossing and targeting cancer cells with an increase of liposome uptake; **(C)** Theranostic with labeled liposomes.

In this study, we propose liposomes labeled with the radionuclide fluorine-18, exploiting the “surface radiolabeling” approach, with a radiolabeling performed on liposome formulations functionalized with mApoE and an MSLP (Scheme 4). The liposome surface was modified with mApoE to increase brain penetration and tumor cell uptake by low-density lipoprotein receptor (LDL-R) targeting (Pawar et al., 2021) and with MSLP to confer an MMP-dependent drug release. This liposome formulation has already been used in other studies, where liposomes successfully crossed the BBB *in vitro* and efficiently released an encapsulated fluorescein dye when exposed to MMP-2 and MMP-9, demonstrating their potential to deliver payloads to the brain (Bana et al., 2014; Giofrè et al., 2022).

### 4.1 Liposome formulation dimension after radiosynthesis

As shown in Section 3.3.2, the m_A_-DOPE-functionalized liposome surface was radiolabeled with **[** ^**18**^ **F]3** via the CuAAC reaction, yielding **[** ^**18**^ **F]C-Lip** with good radiochemical yield and purity (RCY not corrected for decay ∼10%, radiochemical purity >95%, and total radiosynthesis time: 126 min) and with suitable physicochemical features (size <200 nm, PDI <0.2, and charge −25.3 ± 3.5 mV), showing that liposome size was not affected by reaction conditions.

Based on these positive results, radiolabeling via the CuAAC approach was then carried out on m_A_-Lip-MSLP-mApoE; although radiochemical yields and purity levels were comparable, an undesired increase in the average size of liposomes (diameter ≥500 nm) was noted. Since, as mentioned above, we did not observe such behavior with non-functionalized liposomes, we tentatively attributed the observed phenomenon to the known ability of mApoE to bind metals (Zhang et al., 2022), such as copper. However, this issue deserves further investigation.

The abovementioned hypothesis was further confirmed by the experimental results obtained after the radiolabeling of the m_CA_-DOPE liposome formulation via the CyOctC (copper-free cycloaddition) approach with and without functionalization with mApoE. As shown in Section 3.3.3, **[** ^**18**^ **F]D-Lip** was obtained with lower radiochemical yields (∼5%), but the size of the liposomes was not affected (diameter <200 nm, PDI <0.2, and charge < −30 mV), and the same outcome was observed in the case of the radiolabeling of mApoE- and MSLP-functionalized m_CA_-DOPE liposomes **([** ^**18**^ **F]E-Lip)**.

In summary, the CuAAC approach provided higher radiochemical yields (approximately twice those of the CyOctC approach) and allowed for milder reaction conditions, as the cycloaddition reaction was conducted at room temperature. However, the higher reaction efficiency was counterbalanced by the observed increase in liposome size in the case of protein-functionalized liposomes (mApoE and MSLP). For this reason, the CyOctC approach was considered to be preferable.

### 4.2 Radiolabeled liposome formulation stability in solution and human plasma

The stability of **[** ^**18**^ **F]C-Lip**, **[** ^**18**^ **F]D-Lip**, and **[** ^**18**^ **F]E-Lip** in the final formulated solution and human plasma was monitored up to 4 h after the end of the radiosynthetic procedure. Initially, monitoring was performed using TLC and radio-TLC with 90:10 water/ACN as the eluent (Supplementary Figures S12, 16, 18, 19). Under these conditions, **[** ^**18**^ **F]C-Lip** (as well as **[** ^**18**^ **F]D-Lip** and **[** ^**18**^ **F]E-Lip**) has an R_f_ value of 0, and **[** ^**18**^ **F]3** has an R_f_ value of 0.4. The drawback of this method is that only small organic molecules (such as **[** ^**18**^ **F]3**) can run in the TLC strip, while intact radiolabeled nanoparticles (**[** ^**18**^ **F]C, D,** and **E-Lip**) and possible liposome degradation by-products cannot, as also confirmed by testing the above samples with different solvent concentrations.

Thus, the stability was indirectly evaluated by monitoring, in parallel, the integrity of the radiolabeled liposome by measuring liposome size, PDI, and charge on the one hand and the stability of the radiolabeled m_A_/m_CA_ DOPE (**[** ^**18**^ **F]4** and **[** ^**18**^ **F]5**, see Figure 1) after liposome bilayer disruption by organic solvents (chloroform/ethanol, see the Experimental Section), using radio-TLC with 75:25 DCM/MeOH as the eluent, on the other hand.

Using the abovementioned TLC conditions, the results showed that up to 4 h, only one spot at R_f_∼0.8 was detected due to **[** ^**18**^ **F]4** and **[** ^**18**^ **F]5** (Supplementary Figure S20), as identified by the respective reference standards. Furthermore, the method also allows the monitoring of the possible extent of defluorination, as free fluorine-18 can be detected with R_f_ = 0. At the same time, liposome size, PDI, and charge were measured at 4 h using the same intact samples used before disruption for radio-TLC analysis, and the results confirmed the integrity of the liposome structure.

In the case of **[** ^**18**^ **F]E-Lip**, stability was also evaluated in human plasma after 2 and 4 h of incubation, but the results were less clear, showing other spots on the TLC strip in addition to the expected **[** ^**18**^ **F]5**, including a spot at R_f_ = 0, that was initially attributed to free fluorine-18. However, considering that the intensity of this spot remained stable after 4 h of incubation (Supplementary Figures S21, S22), we support the hypothesis that it is due to fluorinated by-products generated during the liposome disruption procedure in the presence of mApoE and MSLP. Thus, we may conclude that all the considered radiolabeled liposome formulations are stable in solution and plasma, and their stability was not affected by the different radiolabeling approaches.

In summary, as mentioned above, both CuAAC and CyOctC approaches demonstrated comparable results in terms of stability in physiological solution and plasma up to 4 h of incubation. Less clear results were obtained with the protein-functionalized liposome formulation, but it was sufficiently stable to be used for preclinical studies.

### 4.3 Comparison between liposome formulation biodistribution

Kinetics data indicate that all formulations rapidly reach intrathecal tumor regions. Moreover, in the presence of brain-targeting functionalization (mApoE), **[** ^**18**^ **F]E-Lip** revealed a fast clearance rate and a lower uptake in peripheral regions and healthy brain tissue, respectively, thus protecting non-pathological tissues from potential off-target delivery. On the other hand, the presence of the peptide mApoE did not increase the absolute uptake of **[** ^**18**^ **F]E-Lip** in the tumor compared to the other formulations, likely due to the modifications in BBB integrity at the tumor site (Steeg, 2021), resulting in a predominance of the enhanced permeability and retention (EPR) (Shi et al., 2020) effect over targeting. However, as stated, double functionalization (mApoE and MSLP) appears to significantly reduce the uptake of **[** ^**18**^ **F]E-Lip** (0.14 ± 0.06 SUV mean at 120 min, Figure 3b) in the contralateral normal brain parenchyma, as indicated by the absolute uptake values compared to **[** ^**18**^ **F]D-Lip** and **[** ^**18**^ **F]C-Lip** (**0.25 ± 0.07 and 0.2 ± 0.1 SUV mean at 120 min, respectively; **p < 0.01 vs. **[** ^**18**^ **F]E-Lip**; Figures 2b, 3b).

Regarding the tumor region, the lack of significant differences between mApoE-functionalized and non-functionalized nanoparticles is in line with a recent study described by Pizzocri et al. (2021), in which the authors observed a significant enhancement of BBB crossing and therapeutic efficacy of mApoE-functionalized nanoparticles only after radiotherapy (RT) treatment, which was not included in our experimental protocol.

According to this study, the abovementioned effect is associated with an overexpression of the low-density lipoprotein receptor (LDLR, Scheme 4) in both BBB and tumor tissue post-RT, with a consequent increase in functionalized (mApoE) nanoparticle uptake. However, the presence of MMP-sensitive peptide on the **[** ^**18**^ **F]E-Lip** surface does not compromise brain tumor uptake, thus encouraging future studies in order to evaluate the drug release efficacy of the abovementioned liposome formulation.

As shown in the results, comparing the *in vivo* results, the choice between the two radiolabeling approaches (CuAAC or CyOctC) did not significantly affect the uptake in peripheral tissues, brain tumors, and parenchyma. In contrast, the main differences in uptake occurred between protein-functionalized liposomes (**[** ^**18**^ **F]E-Lip**) and non-functionalized liposomes.

A potential limitation of our study concerns the short half-life of fluorine-18, which may limit long-term kinetic studies. Fluorine-18 was selected because it is available by cyclotron production, and its organ distribution, in the case of dissociation from nanoparticles following metabolic degradation, does not affect regions known to accumulate nanoparticles, such as the liver. On the contrary, other radionuclides, such as copper-64, which has a longer half-life than fluorine-18, are known to potentially accumulate in the liver, with tissue retention influenced by the transchelation reaction (Shi et al., 2020). *In vivo* biodistribution studies using copper-64 and classical macrocyclic chelators such as 1,4,7,10-tetra-aza-cylcododecane-*N, N′, N″, N*‴-tetraacetic acid (DOTA) (Rogers et al., 2003; Rogers et al., 2004) showed *in vivo* stability issues, which can result in high uptake of activity by non-target tissues such as the liver (Rogers et al., 2003). Despite the limited duration of the study, at 180 min post-injection, radioactivity uptake was reduced or had plateaued in all examined tissues, indicating that fluorine-18 was sufficient to describe the regional tissue distribution/retention of nanoparticles. However, the results showed that the time frame evaluated using fluorine-18 was sufficient to visualize and measure the kinetics of nanoparticle distribution in the brain and periphery after a single i.v. administration.

## 5 Conclusion

In this study, two liposome formulations containing modified DOPE were successfully prepared and allowed for fluorine-18 radiolabeling directly on the liposome surface via both CuAAC “click” chemistry and “copper-free” CyOctC reactions. Radiochemical yields, purity, and stability in the final formulation and plasma were suitable for the preclinical evaluation of the nanoparticles and potential clinical applications. The radiosynthetic procedures were fully automated, prompting a general-purpose method for the radiolabeling of liposomes. Furthermore, the *in vivo* biodistribution in an orthotopic mouse model of glioma (Gli36ΔEGFR cell line) was assessed by PET/CT, using fluorine-18-radiolabeled liposomes either functionalized with mApoE/MSLP proteins **([** ^**18**^ **F]E-Lip**) or without functionalization (**[** ^**18**^ **F]C-Lip** and **[** ^**18**^ **F]D-Lip**), indicating that all formulations rapidly reached intrathecal tumor regions. Moreover, in the case of the targeted **[** ^**18**^ **F]E-Lip**, despite the lack of improvement in tumor uptake, a generally fast clearance rate in peripheral regions and a lower uptake in healthy brain tissue were noted, thus reducing the off-target delivery of the drug when nanoparticles are used as carrier agents.

## Data Availability

The datasets presented in this study can be found in online repositories. The names of the repository/repositories and accession number(s) can be found in the article/Supplementary Material.
