# Supplementary material for: Automated fluorine-18 radiolabeling via an alkyne–azide cycloaddition reaction on a dual peptide-functionalized liposome surface for in vivo PET imaging
Source: Front Pharmacol. 2025 Apr 28;16:1566257. doi: 10.3389/fphar.2025.1566257 (PMC12066565; doi:10.3389/fphar.2025.1566257)
Supplement: Supplementary file 1 [file DataSheet1.pdf]

Supporting Information

Automated fluorine-18 radiolabeling via an alkyne-azide cycloaddition reaction on a dual peptide-functionalized liposomes surface for PET imaging

Marco Iannone*, Marcelo Kravicz*, Paolo Rainone*, Antonia I. Antoniou*, Stefano Stucchi, Silvia Valtorta, Arianna Amenta, Elia Anna Turolla, Sara Pellegrino, Daniele Passarella, Elisa Vino, Sergio Todde, Francesca Re, Pierfausto Seneci, Rosamaria Moresco

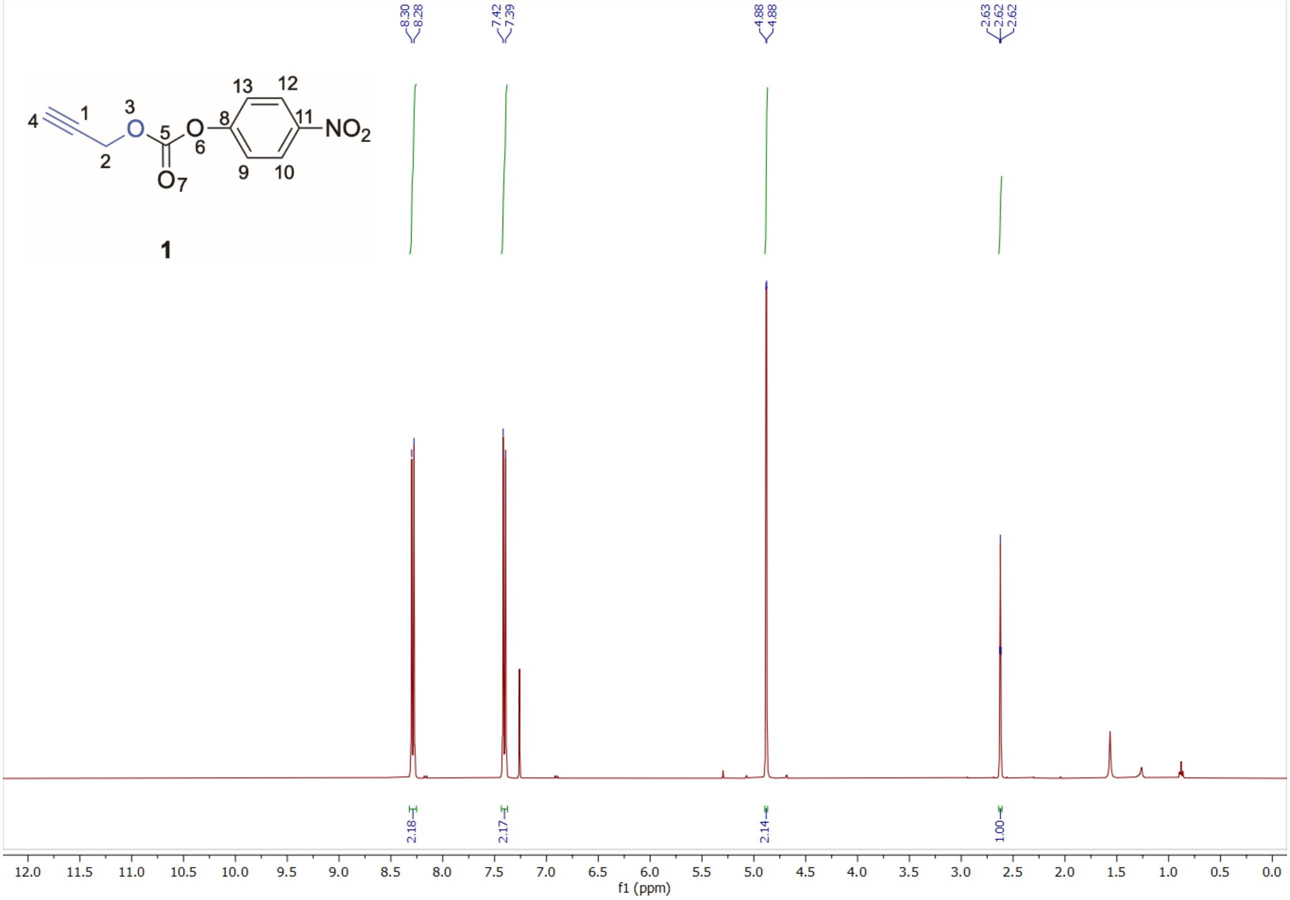

Figure S1. ^{1}H NMR of 4-nitrophenyl prop-2-yn-1-yl carbonate (**1**).

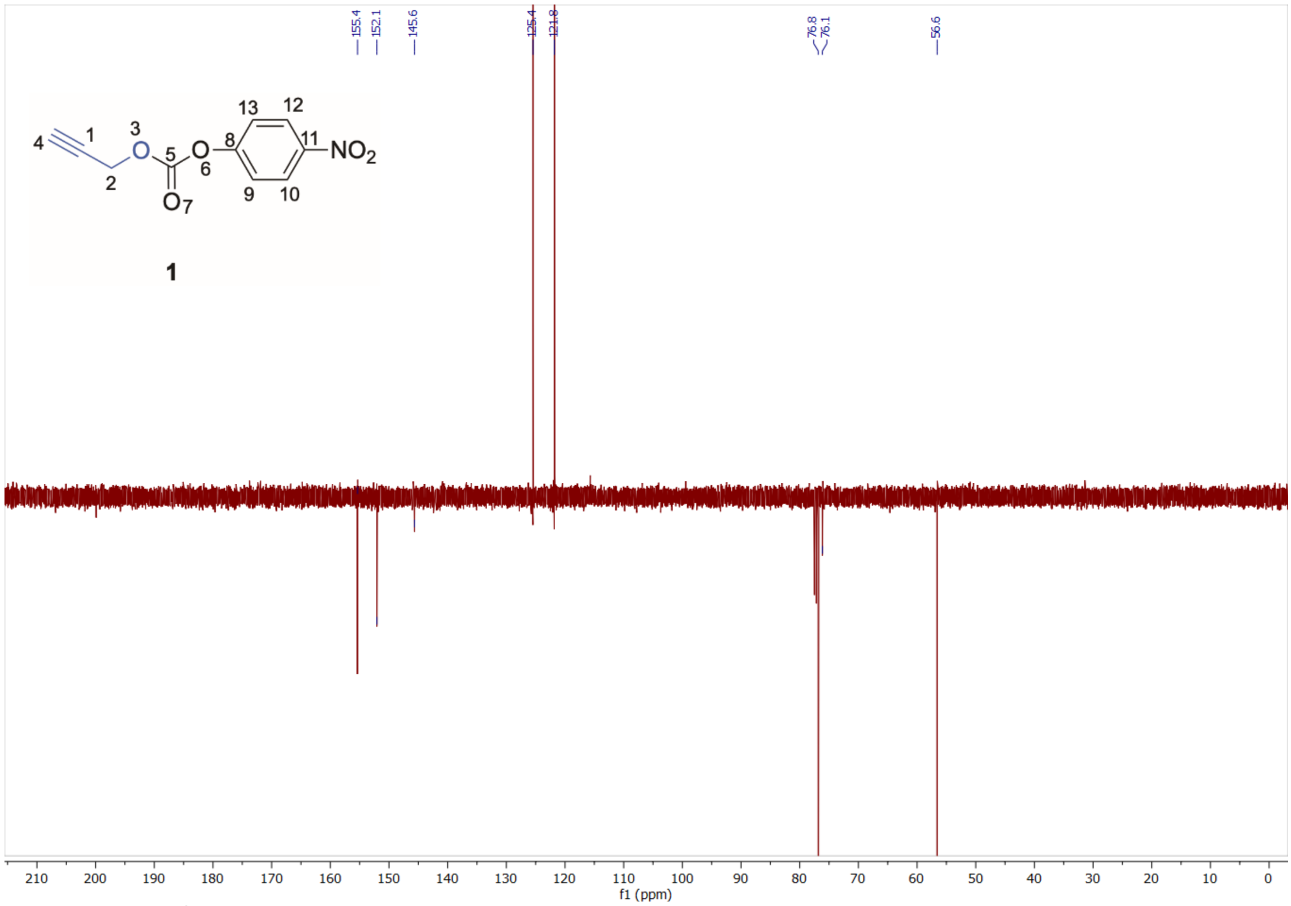

Figure S2. ^{13}C NMR of 4-nitrophenyl prop-2-yn-1-yl carbonate (**1**).

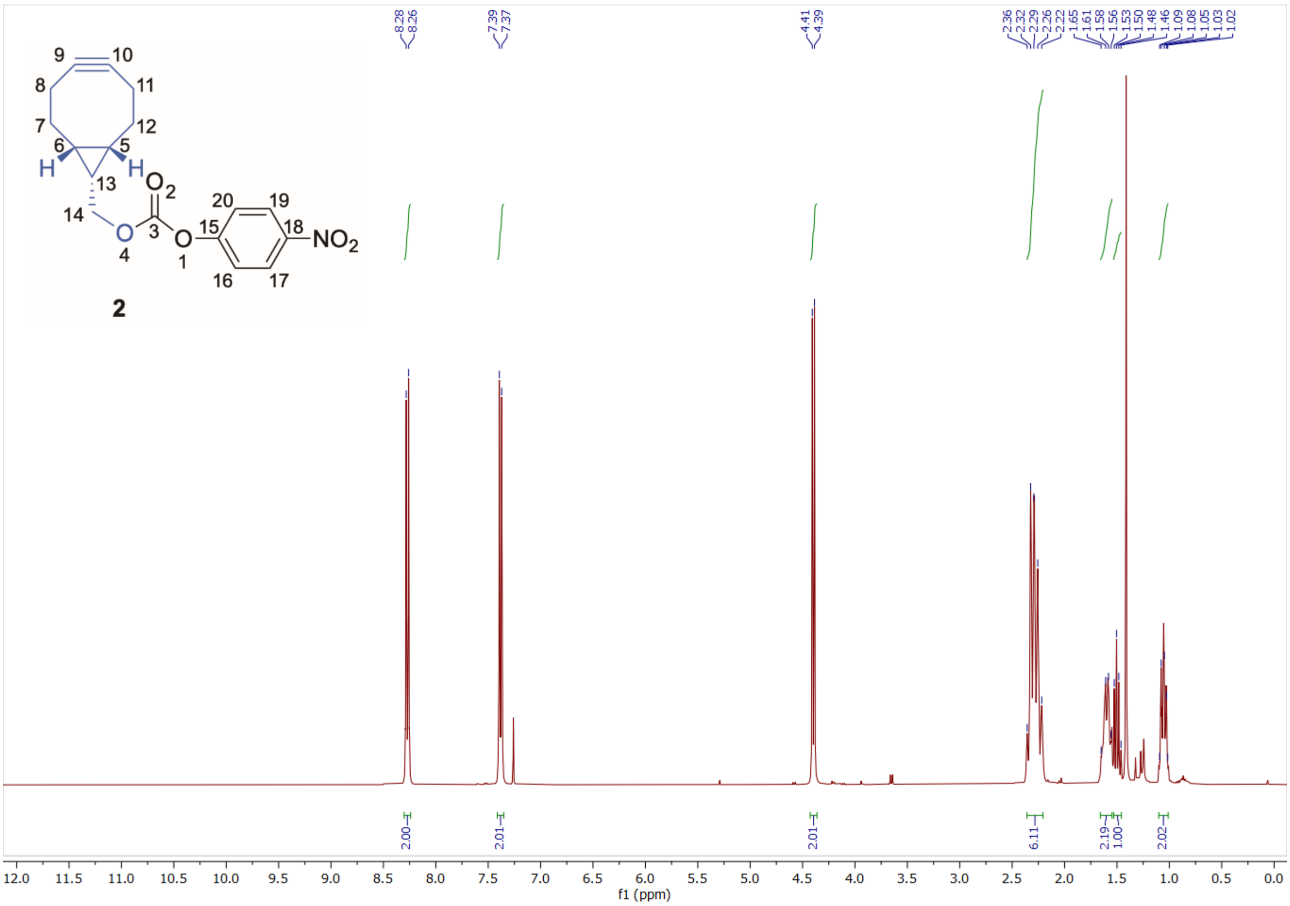

Figure S3. ^{1}H NMR of ((1R,8S,9S)-Bicyclo[6.1.0]non-4-yn-9-yl)methyl 4-nitrophenyl carbonate (**2**).

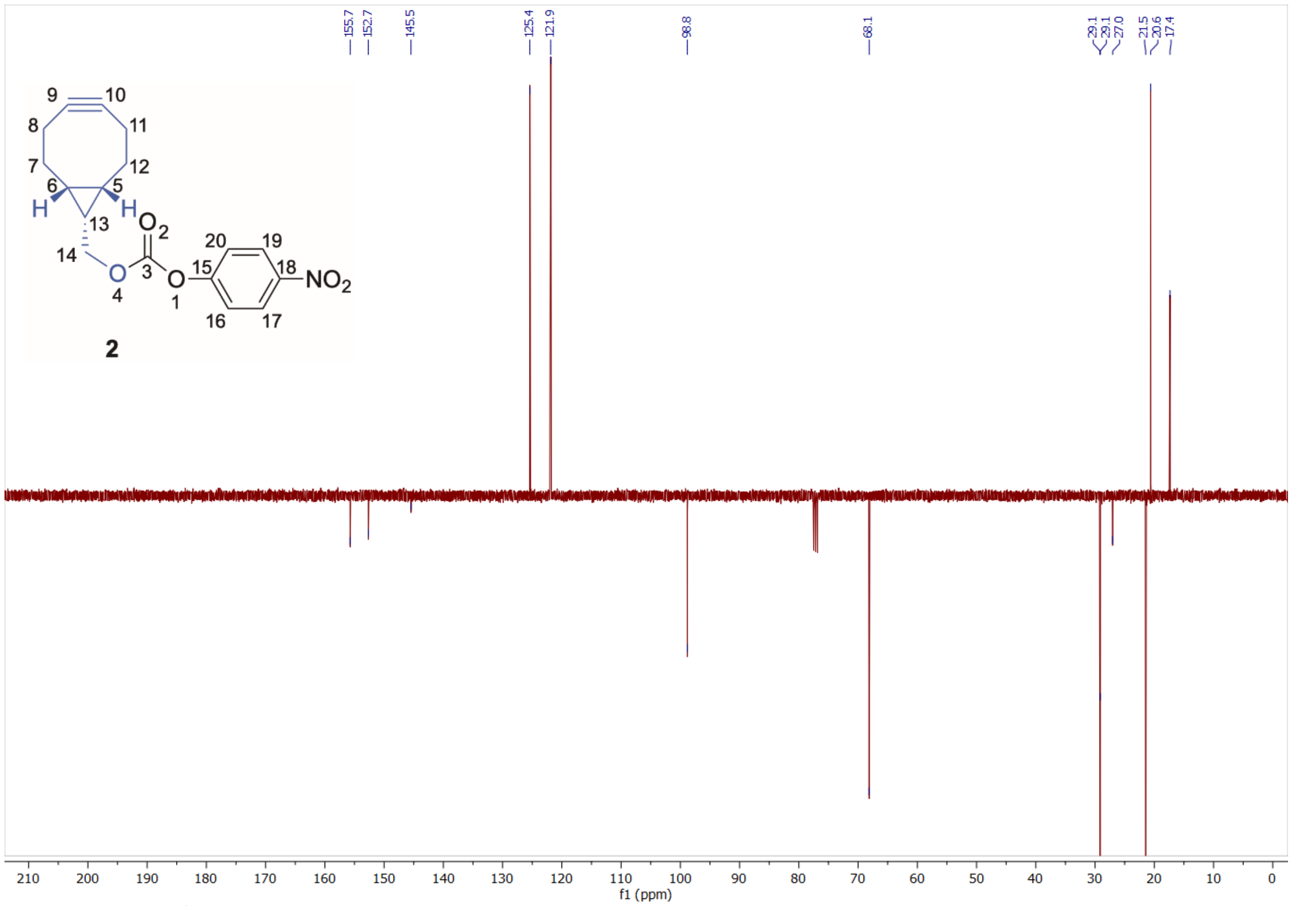

Figure S4. ^{13}C NMR of ((1R,8S,9S)-Bicyclo[6.1.0]non-4-yn-9-yl)methyl 4-nitrophenyl carbonate (**2**).

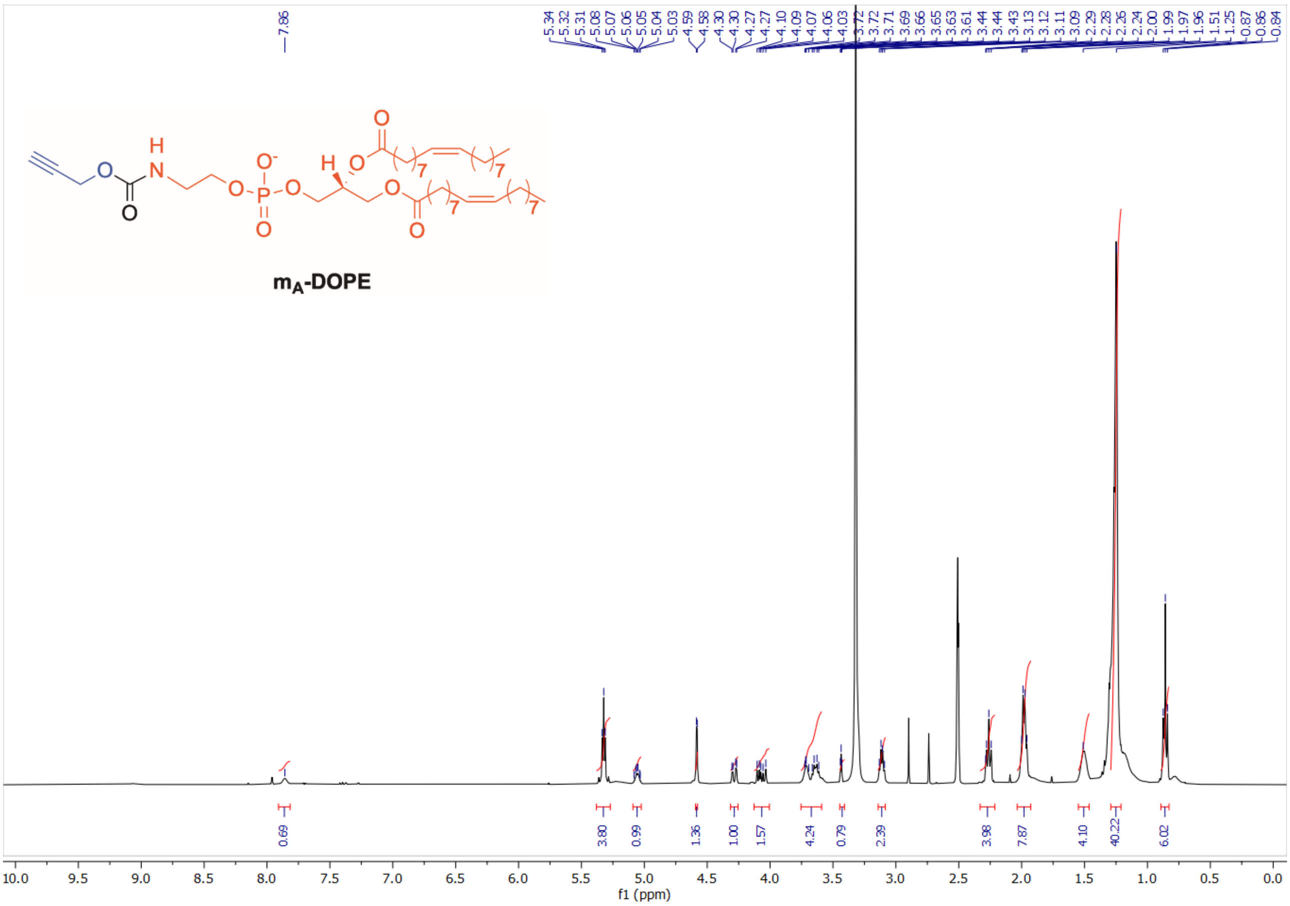

Figure S5. ^{1}H NMR of linear alkyne-bearing carbamate (m$_A$-DOPE).

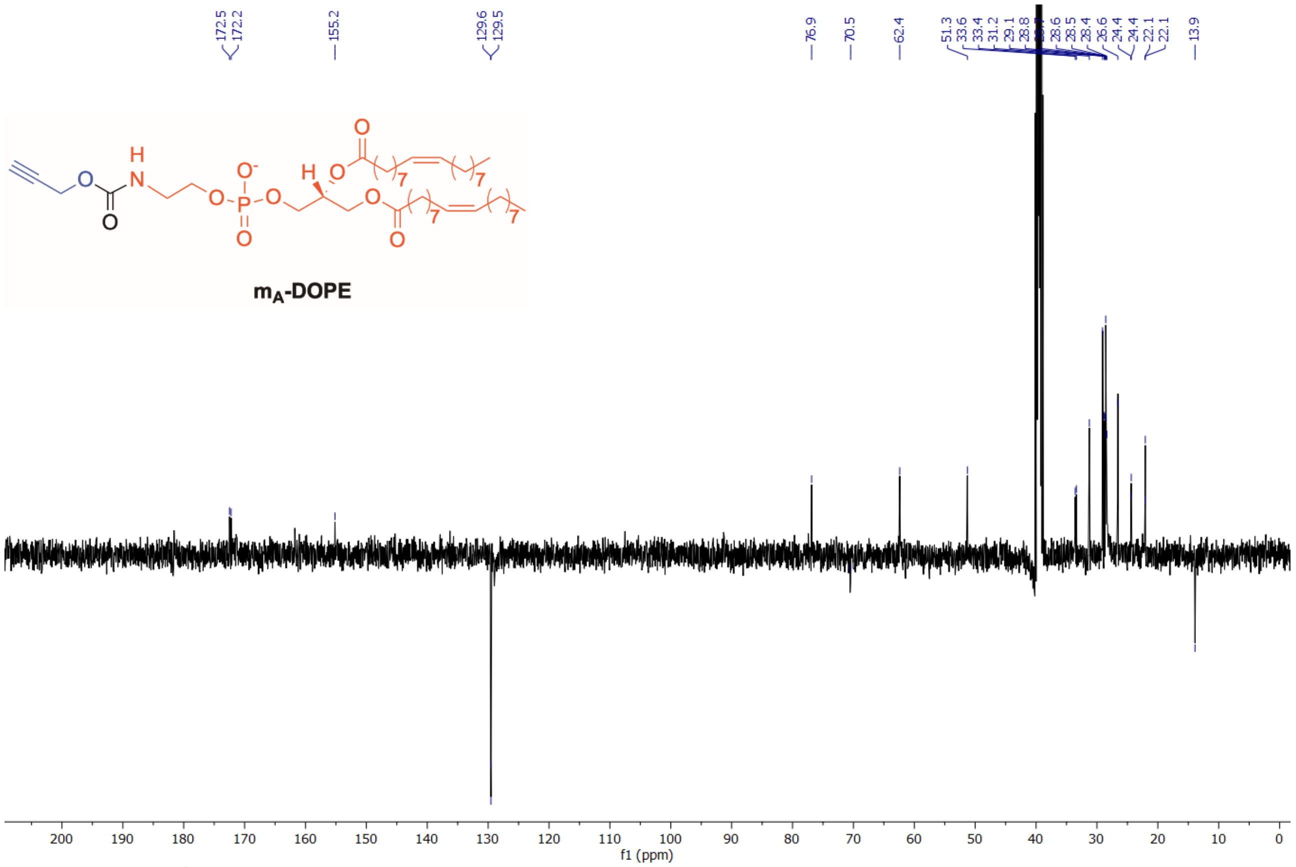

Figure S6. ^{13}C NMR of linear alkyne-bearing carbamate (mA-DOPE).

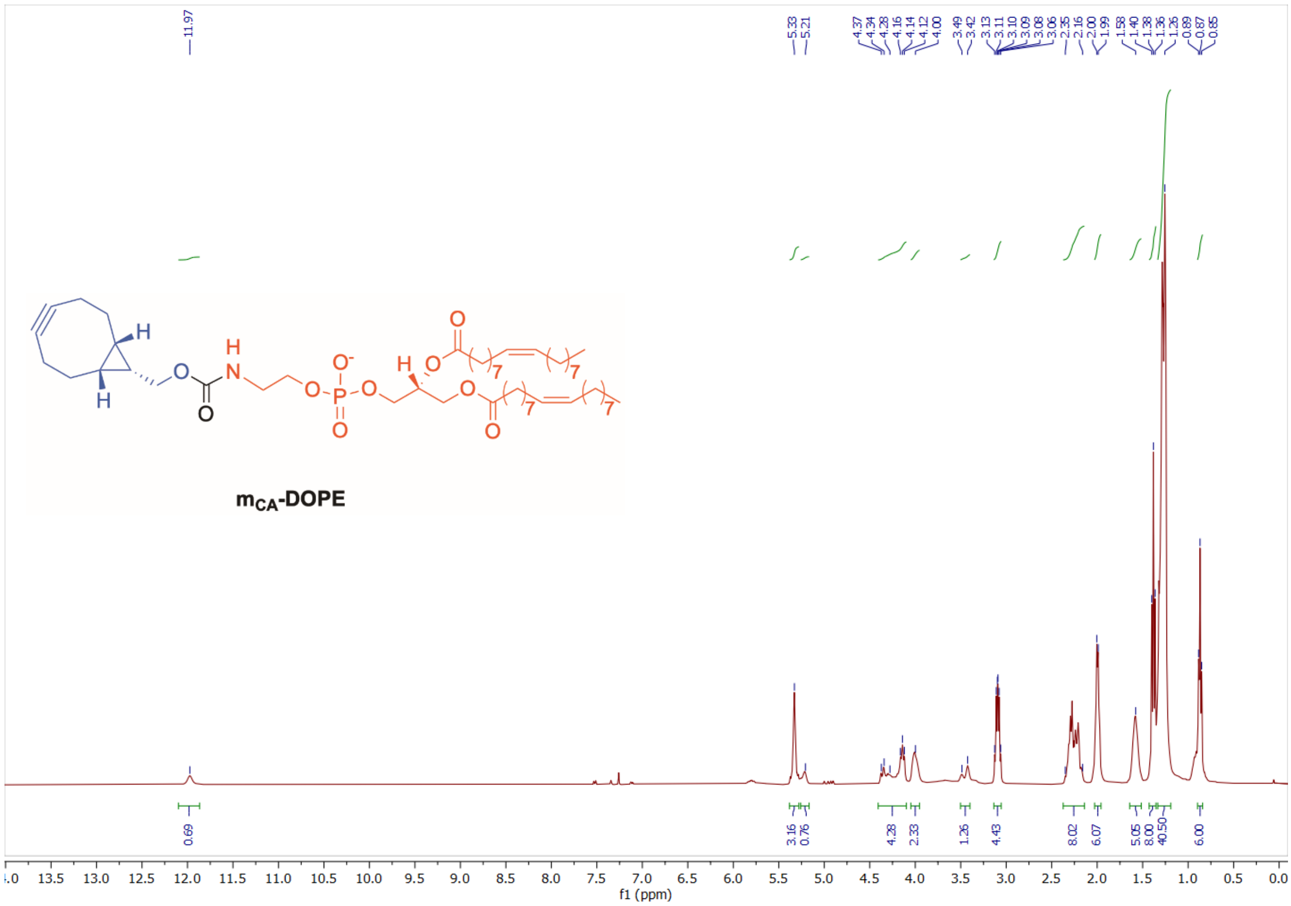

Figure S7. ^{1}H NMR of cyclic alkyne-bearing carbamate (m$_{CA}$-DOPE).

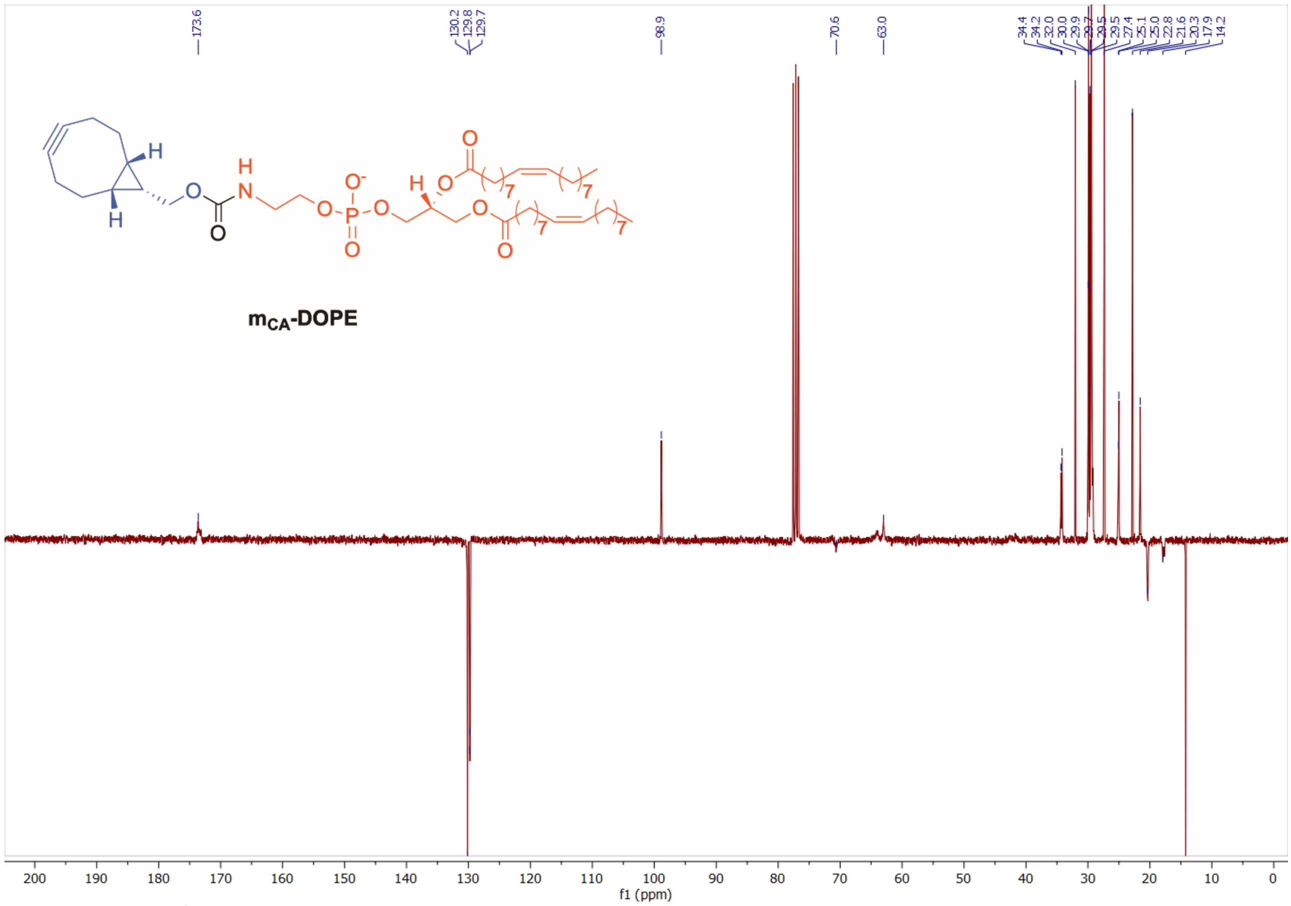

Figure S8. ^{13}C NMR of cyclic alkyne-bearing carbamate (m_{CA}-DOPE).

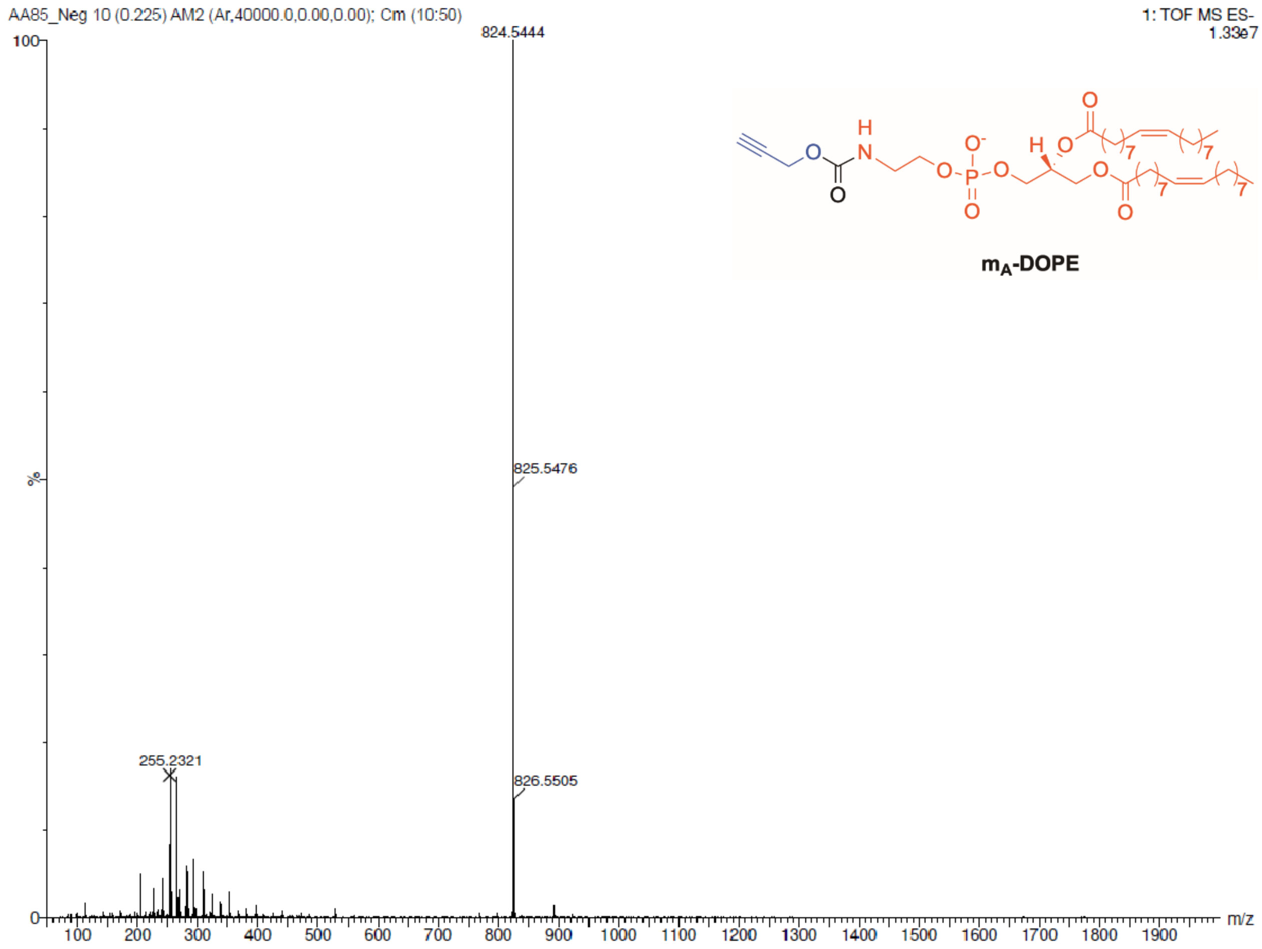

Figure S9. HRMS of cyclic alkyne-bearing carbamate (mA-DOPE).

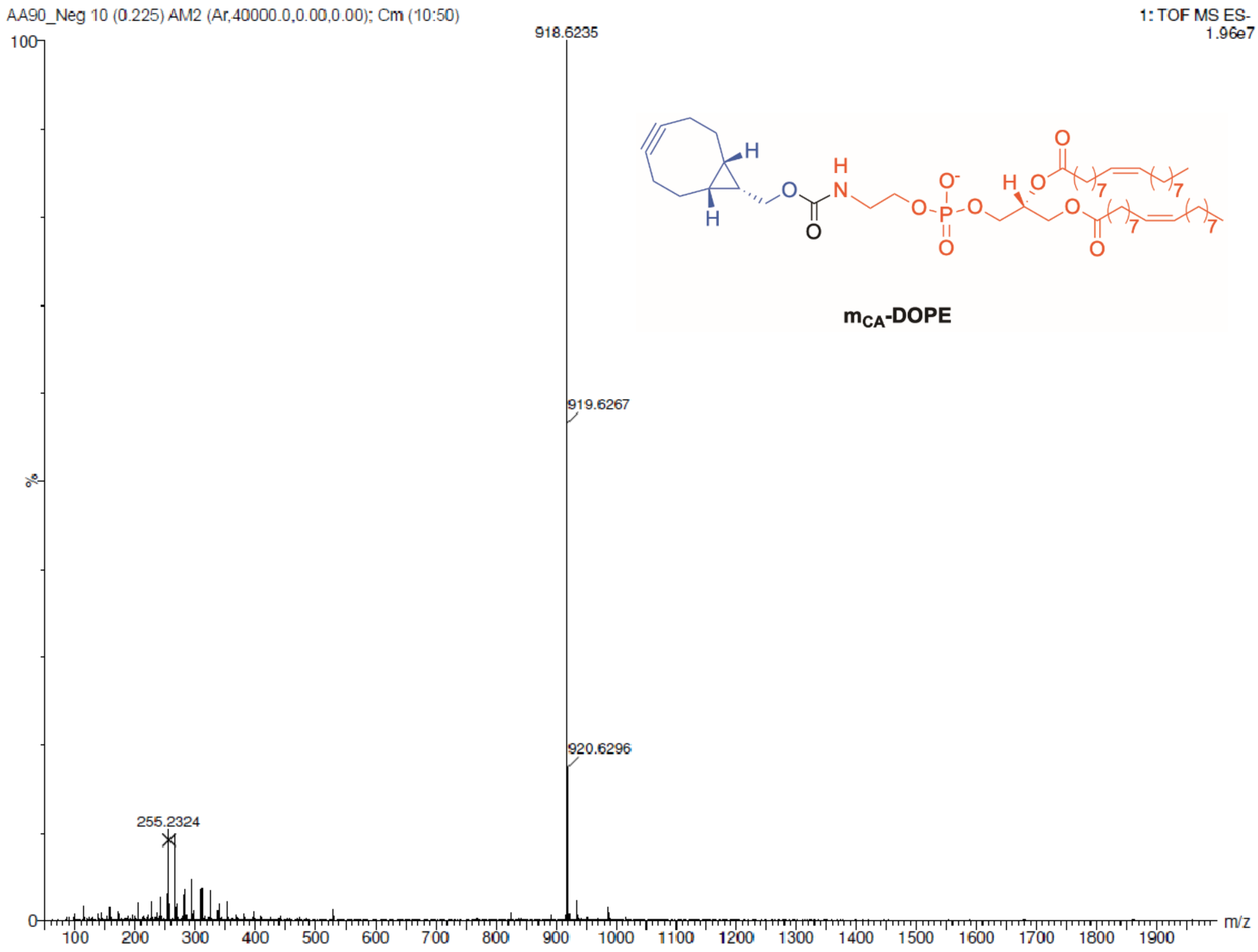

Figure S10. HRMS of cyclic alkyne-bearing carbamate (m_{CA}-DOPE).

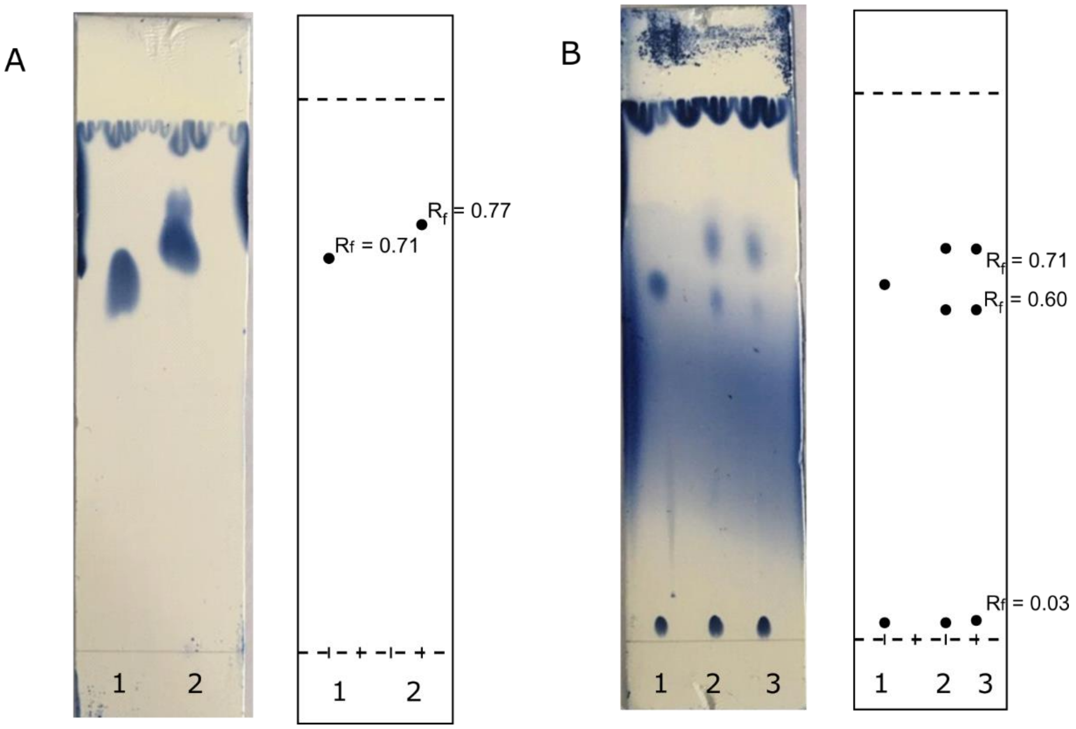

Figure S11. Monitoring of the click reaction on liposome surface by thin layer chromatography (TLC). An aliquot of the liposomal formulation was mixed in chloroform, the aqueous phase was removed, and the collected organic phase was analyzed by TLC using 75:25 (v/v) DCM:MeOH as mobile phase. A) m_A-DOPE (lane 1) and the product **4** (lane 2). B) m_A-Lip CuAAC reaction with [19F]-azide (**3**) after 30 min (lane 2) and 1 h (lane 3) reaction time. m_A-Lip was used as control (lane 1). 75:25 (v/v) DCM:MeOH was used as an eluent mixture for TLC.

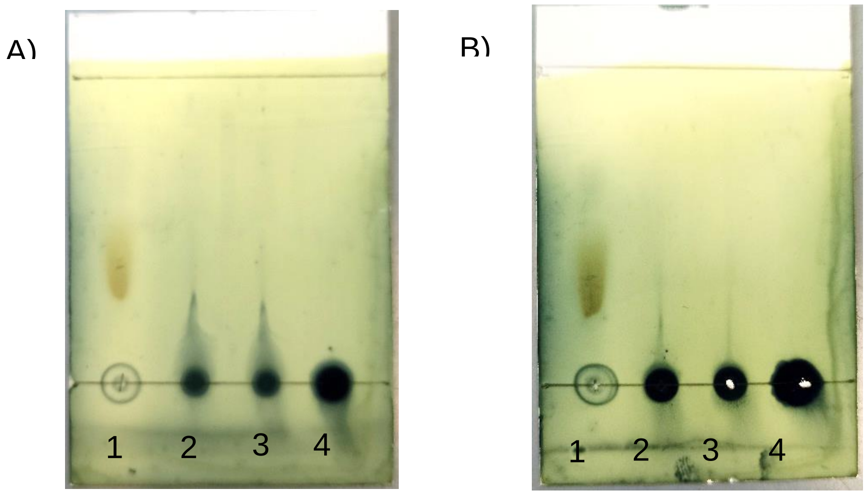

Figure S12. TLC. **A**) $[^{19}F]$F-azide (**3**) (lane 1), m_A-DOPE (lane 2), Product **4** (lane 3); m_A-Lip (lane 4). **B**) $[^{19}F]$F-azide (**3**) (lane 1), m_{CA}-DOPE (lane 2), Product **5** (lane 3); m_{CA}-Lip (lane 4). 90:10 (v/v) water:ACN was used as eluent mixture for all TLCs.

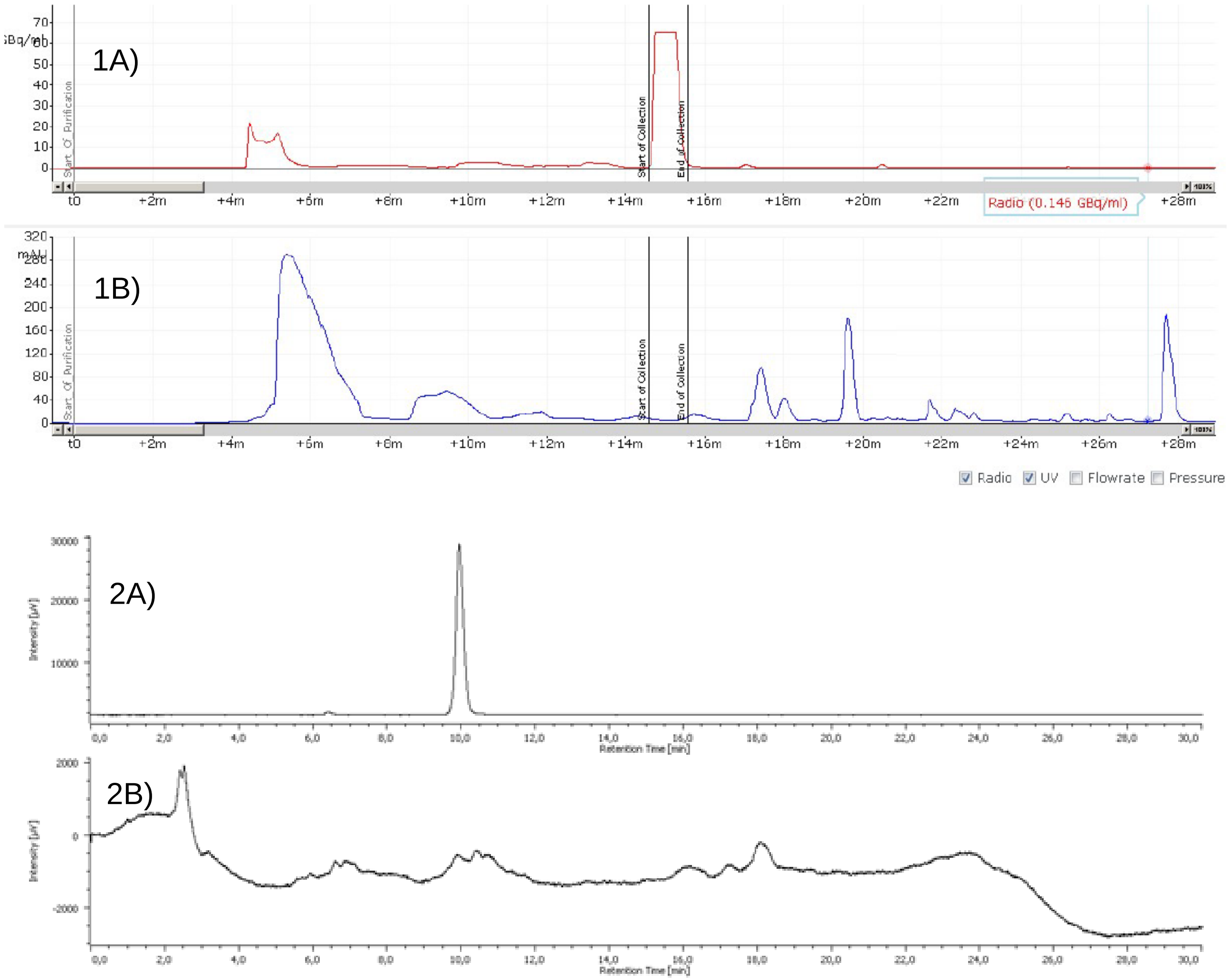

Figure S13. Semi-preparative and analytical HPLC. 1A, 2A: Radiochemical detector. 1B, 2B: UV detector. Purification of **[^{18}F]3** using after semi-preparative HPLC. Semi-preparative RP-HPLC conditions: Clarity Oligo-RP 5µm column, 250x10 mm; water:ACN gradient from 60:40 to 20:80 in 20 min, 5 mL/min, 220 nm, UV detector. Rt: 17 min. Quality control of purified **[^{18}F]3**: Analytical RP-HPLC conditions: XTerra C18 5µm column, 250 × 4.6 mm; water:ACN gradient from 60:40 to 20:80 in 20 min; 1 mL/min, 220 nm, UV detector. Rt: 10.2 min.

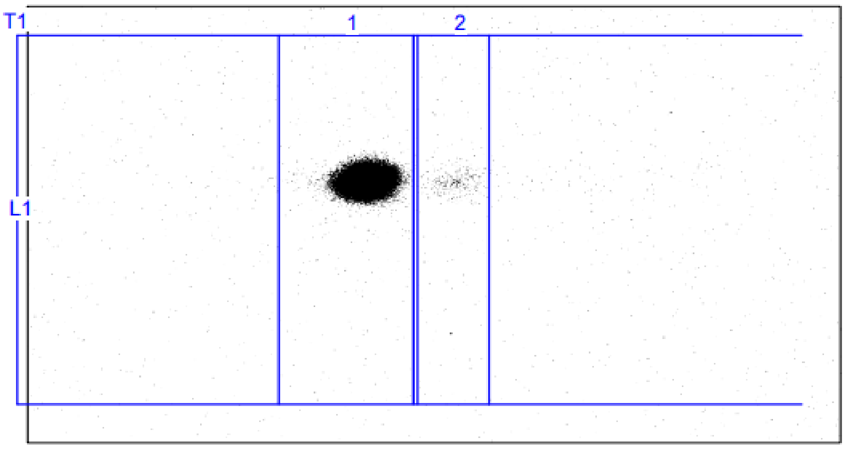

Lane #1

ID	Dist From Origin(mm)	RF	Gross DLU	% Sum	% Max Reg.
**1 - Prof**	41.5	0.4	1,510,740.8	94.4	100.0
**2 - Prof**	52.8	0.5	88,805.3	5.6	5.9
**Lane**			2,025,770.5		
**UnRes**			426,224.4		

Figure S14. Radio-TLC. Purified **[^{18}F]3** after semi-preparative HPLC. Rf= 0.4. Eluent mixture: 90:10 water:ACN

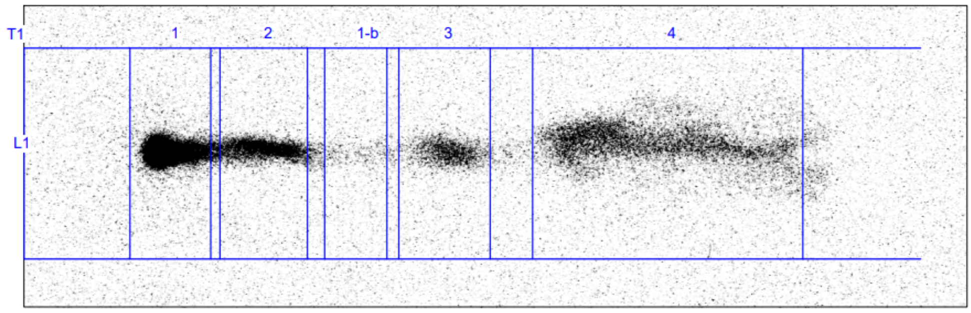

Lane #1

Background Subtraction: Regions = 211.303 DLU /mm2

ID	Dist From Origin(mm)	RF	Gross DLU	Background Subtract	Net DLU	Net % Sum	Net % Max Reg.
1 - Prof	14.8	0.0	1,630,368.7	42,126.6	1,588,242.2	75.9	100.0
2 - Prof	26.4	0.2	199,422.0	45,017.6	154,404.4	7.4	9.7
3 - Prof	46.8	0.4	107,188.6	47,082.6	60,106.0	2.9	3.8
4 - Prof	70.2	0.7	428,358.7	139,182.8	289,175.9	13.8	18.2
1-b - Prof			32,214.4				
Lane			2,552,279.0	462,979.1	2,089,299.9		
UnRes			186,941.0	189,569.5	-2,628.5		

Figure S15. Radio-TLC, showing the crude mixture of **[^{18}F]C-Lip**. Rf=0 liposome (prof-1). Rf=0.4 **[^{18}F]3** (prof-3). Eluent mixture: 90:10 water:ACN

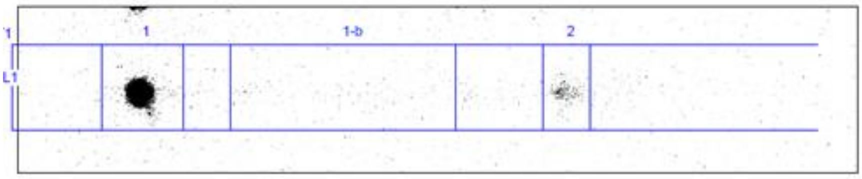

Lane #1
Background Subtraction: Regions = 161.609 DLU /mm2

ID	Dist From Origin(mm)	RF	Gross DLU	Background Subtract	Net DLU	Net % Sum	Net % Max Reg.
1 - Prof	15.5	0.0	1,024,810.9	16,290.6	1,008,520.2	98.3	100.0
2 - Prof	68.9	0.7	27,164.8	9,226.6	17,938.3	1.7	1.8
1-b - Prof			45,123.6				
Lane			1,184,338.7	161,609.0	1,022,729.7		
UnRes			132,363.0	136,091.8	-3,728.8		

Figure S16. Radio-TLC, showing a pure product formulation of **[^{18}F]C-Lip** up to 4h in physiological solution. Rf=0 liposome (prof-1). Rf=0.4 **[^{18}F]3**. Eluent mixture: 90:10 water:ACN.

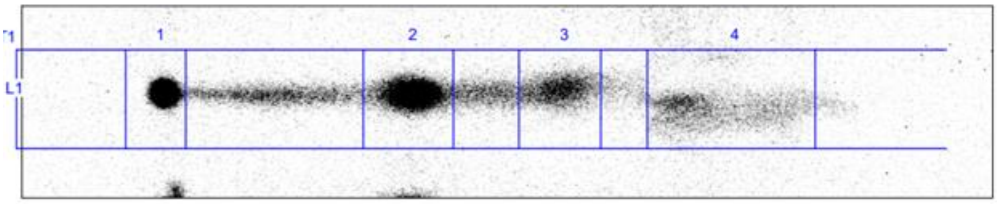

Lane #1

ID	Dist From Origin(mm)	RF	Gross DLU	% Sum	% Max Reg.
1 - Prof	15.5	0.0	746,914.6	40.1	100.0
2 - Prof	42.4	0.4	589,206.0	31.6	78.9
3 - Prof	58.4	0.6	240,222.3	12.9	32.2
4 - Prof	75.5	0.8	287,565.7	15.4	38.5
Lane			2,343,139.6		
UnRes			479,231.0		

Figure S17. Radio-TLC, showing the crude mixture of **[^{18}F]D-Lip**. Rf=0 liposome (prof-1). **[^{18}F]3** (prof-2) Rf=0.4. Eluent mixture: 90:10 water:ACN

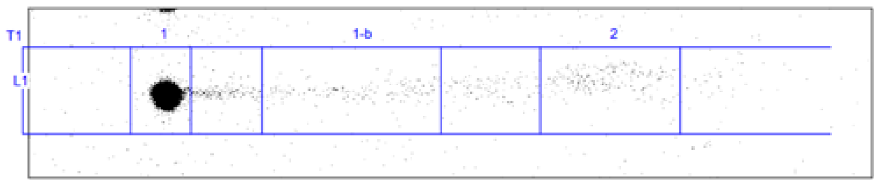

Lane #1
Background Subtraction: Regions = 206.017 DLU /mm2

ID	Dist From Origin(mm)	RF	Gross DLU	Background Subtract	Net DLU	Net % Sum	Net % Max Reg.
1 - Prof	17.3	0.0	687,868.9	15,437.5	672,431.4	98.4	100.0
2 - Prof	72.5	0.8	46,773.7	35,653.3	11,120.5	1.6	1.7
1-b - Prof			45,209.8				
Lane			877,486.3	206,017.1	671,469.2		
UnRes			142,843.7	154,926.3	-12,082.7		

Figure S18. Radio-TLC. Pure product **[18F]D-Lip** up to 4h in physiological solution. Rf=0 liposome (prof-1). Rf=0.4 [18F]3. Eluent mixture: 90:10 water:ACN

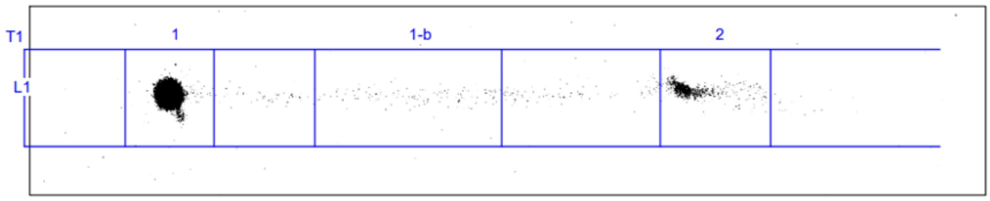

Lane #1
Background Subtraction: Regions = 564.220 DLU /mm2

ID	Dist From Origin(mm)	RF	Gross DLU	Background Subtract	Net DLU	Net % Sum	Net % Max Reg.
1 - Prof	15.3	0.0	2,043,656.4	55,365.1	1,988,291.3	96.1	100.0
2 - Prof	74.6	0.8	148,655.4	67,948.0	80,707.3	3.9	4.1
1-b - Prof			115,260.0				
Lane			2,515,450.0	564,220.4	1,951,229.5		
UnRes			323,138.2	440,907.3	-117,769.1		

Figure S19. Radio-TLC. Pure product **[18F]E-Lip** up to 4h in physiological solution and after 4h in plasma incubation. Rf=0 liposome (prof-1). Rf=0.4 **[18F]3**. Eluent mixture: 90:10 water:ACN

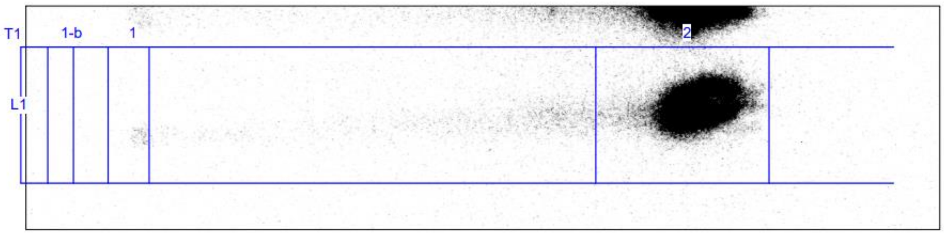

Lane #1

Background Subtraction: Regions = 192.386 DLU /mm2

ID	Dist From Origin(mm)	RF	Gross DLU	Background Subtract	Net DLU	Net % Sum	Net % Max Reg.
1 - Prof	12.7	0.0	26,597.5	13,363.1	13,234.4	0.4	0.4
2 - Prof	76.9	0.8	3,729,722.7	55,973.6	3,673,749.1	99.6	100.0
1-b - Prof			8,320.4				
Lane			4,241,722.0	282,641.6	3,959,080.4		
UnRes			485,401.8	213,304.9	272,096.9		

Figure S20. Radio-TLC. Pure product **[^{18}F]C,D-Lip** up to 4h in physiological solution. Rf=0.8 **[^{18}F] 4** or **5** (prof-2). Eluent mixture: 75:25 DCM:MeOH

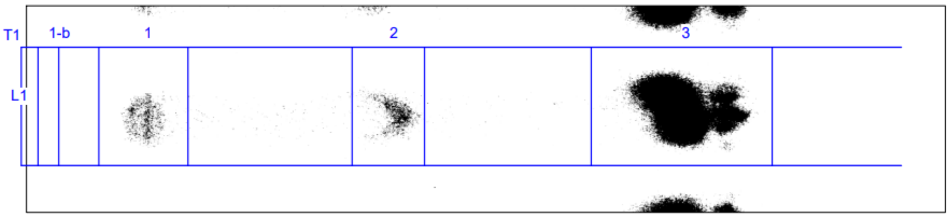

Lane #1

Background Subtraction: Regions = 190.333 DLU /mm2

ID	Dist From Origin(mm)	RF	Gross DLU	Background Subtract	Net DLU	Net % Sum	Net % Max Reg.
1 - Prof	14.2	0.0	393,731.7	24,600.0	369,131.7	6.0	6.8
2 - Prof	41.9	0.4	404,035.6	19,852.6	384,183.0	6.2	7.1
3 - Prof	75.4	0.8	5,485,898.1	49,631.6	5,436,266.5	87.8	100.0
1-b - Prof			5,610.5				
Lane			6,968,912.5	241,900.2	6,727,012.4		
UnRes			685,247.1	147,815.9	537,431.2		

Figure S21. Radio-TLC. Pure product **[^{18}F]E-Lip** up to 2h of incubation in human plasma. Rf=0.8 **[^{18}F] 5** (prof-3). Eluent mixture: 75:25 DCM:MeOH

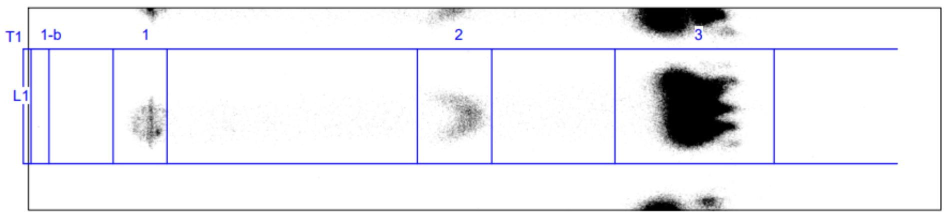

Lane #1
Background Subtraction: Baseline

ID	Dist From Origin(mm)	RF	Gross DLU	Background Subtract	Net DLU	Net % Sum	Net % Max Reg.
**1 - Prof**	13.9	0.0	359,438.0	14,839.3	344,598.7	4.0	4.5
**2 - Prof**	49.8	0.5	545,787.4	20,646.0	525,141.4	6.1	6.8
**3 - Prof**	76.9	0.8	7,762,506.4	43,657.6	7,718,848.8	89.9	100.0
**1-b - Prof**			5,161.5				
**Lane**			9,598,557.2	241,084.8	9,357,472.4		
**UnRes**			930,825.5	161,941.9	768,883.6		

Figure S22. Radio-TLC. Pure product **[^{18}F]E-Lip** up to 4h of incubation in human plasma. Rf=0.8 **[^{18}F] 5** (prof-3). Eluent mixture: 75:25 DCM:MeOH

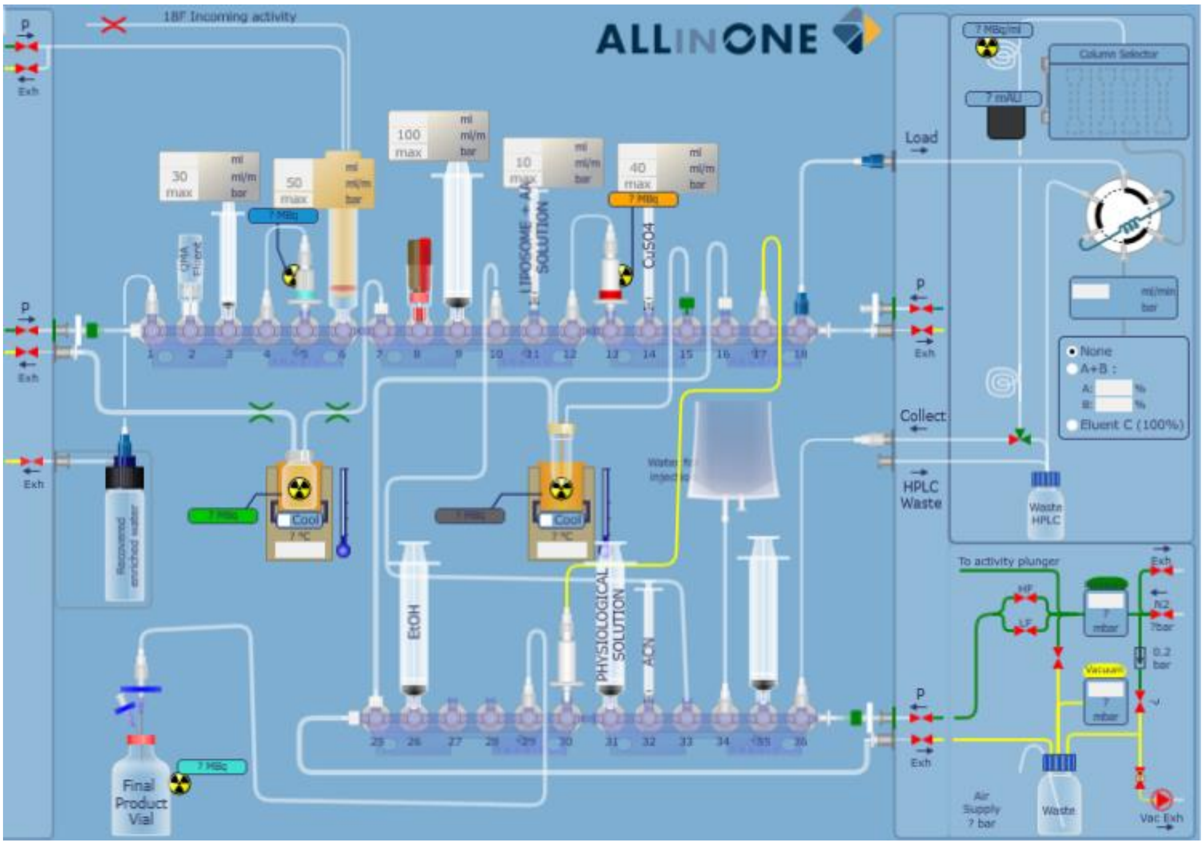

Figure S23. Trasis All-in One layout for a CuAAC-based approach. Position 2: 1 mL of kryptofix solution reservoir. Position 3: aqueous potassium carbonate solution. Position 8: iodoazide precursor (6) solution in 1 mL ACN. Position 11: liposome and ascorbic acid solution. Position 13: tC18 cartridge. Position 14: copper sulfate solution. Position 30: BabyBio^{TM}Dsalt cartridges.

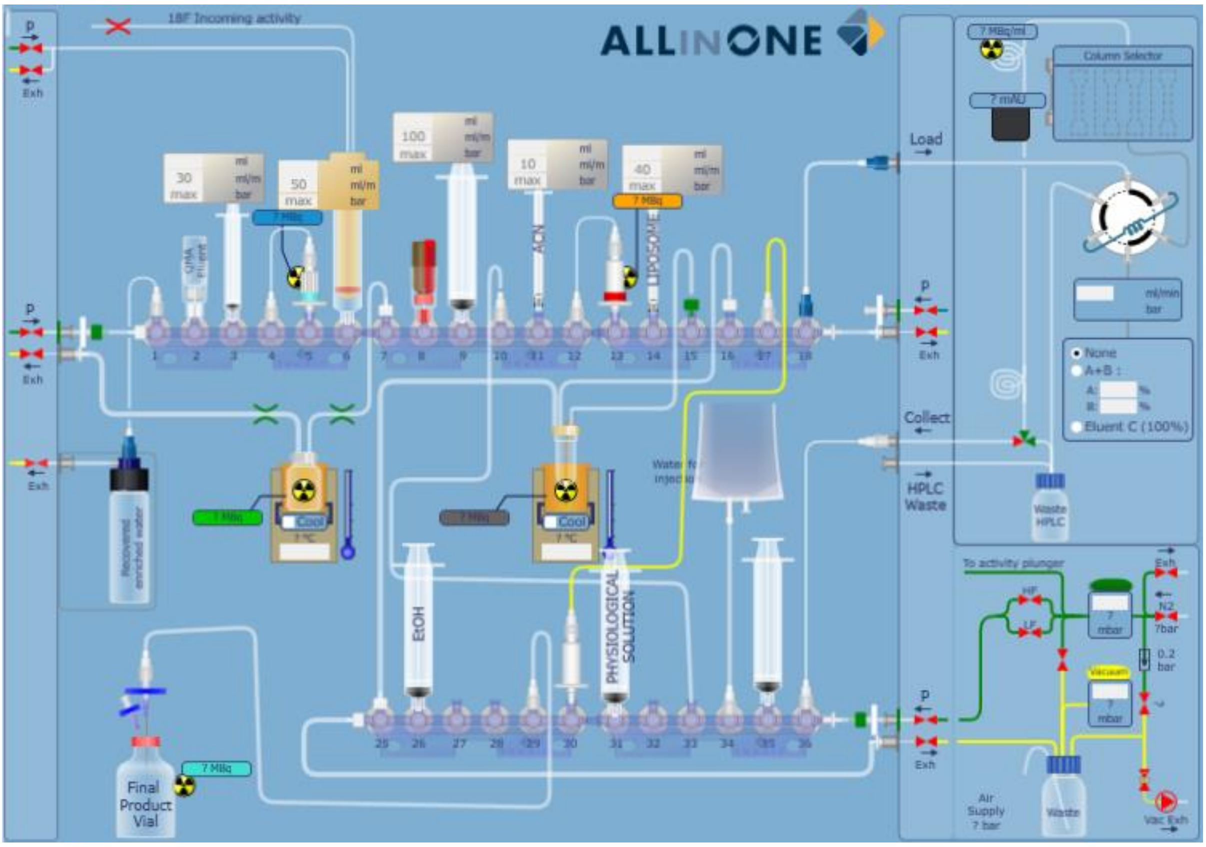

Figure S24. Trasis All-in One layout for a "copper-free" CyOctC-based approach. Position 2: 1 mL of kryptofix solution reservoir. Position 3: potassium carbonate solution. Position 8: iodoazide precursor (6) solution in 1mL ACN. Position 13: tC18 cartridge. Position 14: liposome solution. Position 30: BabyBio^{TM}Dsalt cartridges.

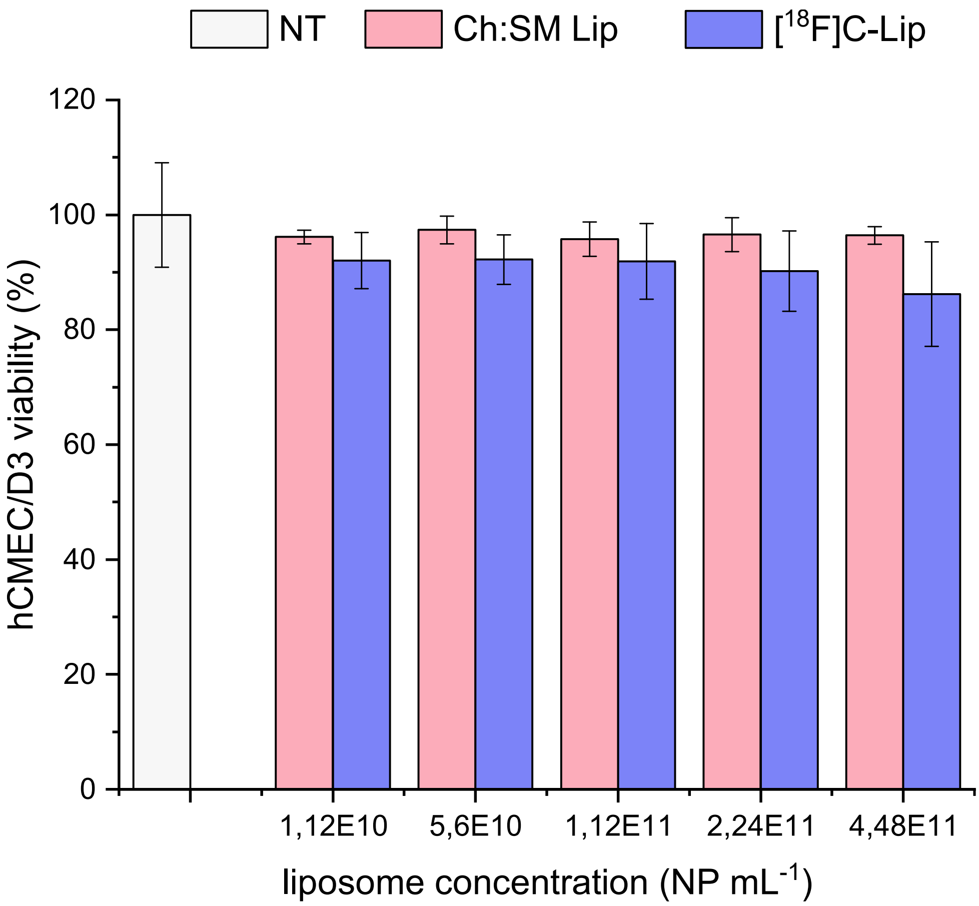

Figure S25. hCMEC/D3 viability after treatment with [^{18}F]C-Lip measured *in vitro*, after complete fluorine-18 decay (48h after radiosynthesis). NT, non-treated cells; ChSM Lip, liposomes without any functionalization
